# Enhanced and Reusable Poly(hydroxy urethane)-Based Low Temperature Hot-Melt Adhesives

**DOI:** 10.1021/acspolymersau.1c00053

**Published:** 2022-01-10

**Authors:** Alvaro Gomez-Lopez, Naroa Ayensa, Bruno Grignard, Lourdes Irusta, Iñigo Calvo, Alejandro J. Müller, Christophe Detrembleur, Haritz Sardon

**Affiliations:** † POLYMAT and Department of Polymers and Advanced Materials: Physics, Chemistry and Technology, Faculty of Chemistry, University of the Basque Country UPV/EHU, Paseo Manuel de Lardizabal 3, 20018 Donostia-San Sebastián, Spain; ψ Center for Education and Research on Macromolecules (CERM), CESAM Research Unit, University of Liège, allée du 6 août, Building B6A, Agora Square, 4000 Liège, Belgium; ¥ ORIBAY Group Automotive S.L. R&D Department, Portuetxe bidea 18, 20018 Donostia-San Sebastián, Spain; γ IKERBASQUE, Basque Foundation for Science, Plaza Euskadi 5, 48009 Bilbao, Spain

**Keywords:** non-isocyanate polyurethanes, poly(hydroxy urethane)s, hot-melt, adhesives, sustainability, green chemistry

## Abstract

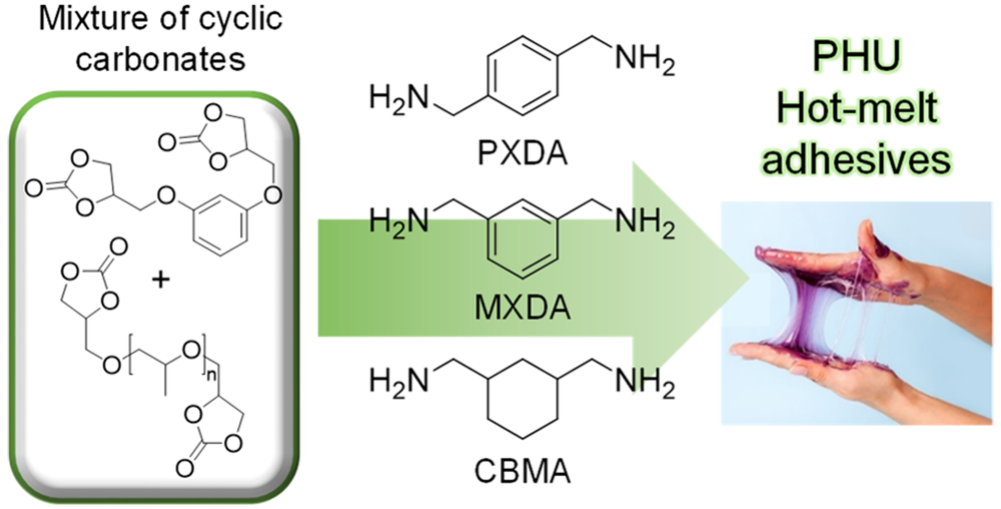

Poly(hydroxy urethane)s (PHUs) based on 5-membered cyclic carbonates have emerged as sustainable alternatives to conventional isocyanate-based polyurethanes. However, while from the point of view of sustainability they represent an improvement, their properties are still not competitive with conventional polyurethanes. In this work, the potential of PHUs as reversible hot-melt adhesives is discussed. We found that with a judicious choice of reagents (i.e., the dicyclic carbonate and diamine), the detrimental hydrogen bonding between the soft segment of the chains and the pendant hydroxyl groups was partially avoided, thus imparting PHUs with hot-melt adhesion properties (i.e., adhesion at elevated temperatures and cohesiveness at a temperature lower than *T*_g_/*T*_m_). The importance of a balanced hard to soft segment ratio, along with the relevance of the chain extender in the final properties, is highlighted. Addition of aliphatic diamines (HMDA, 1,12-DAD) resulted in rubbery materials, while the employment of cycloaliphatic (CBMA) or aromatic ones (MXDA, PXDA) led to materials with hot-melt adhesive properties. The thermoreversibility of all compositions was assessed by rebonding specimens after lap-shear tests. Lap-shear strength values that were comparable to the virgin adhesives were observed. The breaking and reformation of hydrogen bonding interactions was demonstrated by FTIR measurements at different temperatures, as well as by rheological frequency sweep experiments. In order to mitigate the negative impact of the low molar mass PHUs and to enhance the service temperature of the adhesives, a hybrid PHU was prepared by adding a small amount of an epoxy resin, which acts as a cross-linker. These hybrid PHUs maintain the thermoreversibility displayed by thermoplastic PHUs while providing better adhesion at elevated temperatures. We believe that this work provides some important insights into the design of PHU-based hot-melt adhesives.

## 1 Introduction

Hot-melt adhesives (HMAs) are typically solvent-free thermoplastic materials or lightly cross-linked thermosets, which are characterized by their solid state at low temperatures, while presenting low viscosity and good flowing above this temperature.^1^ They provide great bond strengths in short periods upon cooling, which makes them very attractive materials when fast processing is required. Moreover, they are relatively easy to handle, economical, and clean running. Thus, hot-melt adhesives are used in a large range of applications including the automotive industry, packaging, bookbinding, shoe making, textiles, labeling of bottles, disposable products, stamps, and envelopes.^1−3^ Indeed, the global hot-melt adhesive market is projected to reach USD 9.46 billion by 2022.^4^

Typical formulations of HMAs consist of polymers (∼33%), low molar mass resins (∼33%), waxes (∼32.5%), and antioxidants (∼0.5%).^5^ Polymers impart strength and hot tack, resins provide a lower viscosity, improve wettability, and adjust the *T*_g_ of the system, and waxes enhance setting speed and provide heat resistance.^6^ Among the polymers used for HMAs, polyurethanes (PUs) possess high popularity as they show better low temperature properties and greater flexibility than those based on ethylene vinyl acetate and polyamide.^7^ Moreover, they present excellent adhesion on surfaces that are difficult to adhere, such as low surface roughness materials.

PUs are synthesized in two steps. First, an isocyanate-terminated prepolymer is prepared by reacting a long chain polyol with an excess of diisocyanates. The prepolymer is further reacted in a second step with a low molar mass diol, known as a chain extender. The resulting polymer alternates soft segments, mainly containing the polyol, and hard segments, formed through the reaction of the isocyanate and the short chain diols. The incompatibility between the two phases leads to a phase-separated structure consisting of soft and hard domains. This phase separation, which is brought by hydrogen bond-based cross-linking of the hard segment, imparts unique morphological and physical properties.^8^

Nevertheless, traditionally, the synthesis of PUs requires the use of toxic isocyanates, which may provoke asthma and dermatitis.^9−11^ The chemicals employed for the preparation of the isocyanates themselves are also highly toxic, often involving the use of phosgene,^12^ and therefore, issues related to toxicity represent major drawbacks in the use of conventional PUs. To overcome these issues, in the past decade intensive efforts have been directed toward the preparation of PUs using safer and more sustainable approaches that avoid the use of isocyanates.

One of the methods that is considered to be competitive with conventional isocyanate-based PUs is the step-growth polymerization of dicyclic carbonates with diamines. Initially developed in 1957 by Dyer and Scott^13^ as a method to produce polyurethanes avoiding the use of moisture sensitive isocyanates, the current trends for greener chemistries have greatly boosted the development of this chemistry.^14,15^ Indeed, this process for the production of non-isocyanate polyurethanes (NIPUs) is 100% atom economic, and the cyclic carbonate monomers are easily accessible by the facile [3 + 2] chemical insertion of CO_2_ into (natural) epoxy resins.^16−20^ However, the polymerization also has some drawbacks in the preparation of hot-melts.

On the one hand, the ring-opening of the cyclic carbonate generates hydroxyl groups along the polymeric chain, providing the so-called poly(hydroxy urethane)s (PHUs). These hydroxyl groups can establish strong hydrogen bonds with the soft segment, enhancing the miscibility between both phases and suppressing the distinctive phase separation of conventional PUs. Some authors have tackled this issue employing different approaches, selecting carefully the monomers^21−24^ or the synthetic conditions.^25^ However, the resulting polymers showed elastomeric-like behavior, and adhesion performance was not reported. On the other hand, while high molar mass PUs can be easily achieved, in the case of PHUs, the inherent slow aminolysis of the cyclic carbonates and the inter- and intramolecular hydrogen bonding of the PHU chains gives rise to low molar masses, which limits their use as Thermoplastic Polyurethane (TPU) materials.^26^

Recently Sukumaran Nair et al. reported the formation of PHU-based hot-melt adhesives with a thermal transition close to 80–100 °C by reacting aromatic and cycloaliphatic dicyclic carbonates with amino-terminated oligo(propylene glycol).^27^ The authors demonstrated the thermoreversibility of the materials after manually debonding and then rebonding the substrates with no noticeable loss of the adhesion values. While they found a relation between the hydrogen bonding and the thermoreversibility, a rational study to understand the structural needs to design PHU hot-melt adhesives was not provided.

Herein, we aim to provide an in-depth study of the structural features needed to prepare PHU-based HMAs. A series of PHUs was prepared using various hard (resorcinol-based) and soft (PPG-based) dicyclic carbonates and diamines (aliphatic, cycloaliphatic, or aromatic). The influence of the molar composition of blends of dicyclic carbonates, as well as the nature thereof, on the adhesion properties is addressed by rheology, probe tack, lap-shear, shear adhesion failure temperature (SAFT), and static shear resistance measurements. Finally, in order to mitigate limitations arising from the low molar mass PHUs, hybrid PHUs were prepared by combining the non-isocyanate chemistry with the epoxy resin one. The addition of the epoxy resin chemistry endowed PHU hot-melt adhesives with better service temperatures making them valuable alternatives to traditional isocyanate-based PU HMAs.

## 2 Experimental Section

### 2.1 Reagents and Materials

Poly(propylene glycol) diglycidyl ether (*M*_n_ ∼ 640 g mol^–1^) (PPGDGE), Resorcinol diglycidyl ether (RDGE), 1,4-butanediol diglycidyl ether (BDGE), tetrabutylammonium iodide (98%) (TBAI), 1,12-diaminododecane (98%) (1,12-DAD), *p*-xylylenediamine (PXDA) (99%), and hexamethylenediamine (HMDA) (98%) were purchased from Merck KGaA, Germany. 1,3-Cyclohexanebis(methylamine) (*cis* and *trans* mixture) (CBMA) (98%) was purchased from TCI Europe N.V., Belgium. *m*-Xylylenediamine (99%) (MXDA) was purchased from Acros Organics, Belgium. 1,3-Bis(2-hydroxyhexafluoroisopropyl)benzene (97%) (1,3-bis-HFIB) was purchased from Fluorochem, United Kingdom. Solid epoxy resin based on bisphenol A (trade name: D.E.R. 671) was kindly supplied by Oribay Group Automotive S.L., Spain. Deuterated dimethyl sulfoxide (DMSO-*d*_6_) was purchased from Merck KGaA, Germany. The cyclic carbonates used in this study were synthesized by CO_2_ coupling with the commercial precursors using a homemade catalyst reported elsewhere by some of the authors.^28^ All reagents were used without further purification.

Plexiglas XT 20070 [poly(methyl methacrylate) (PMMA), thickness of 3 mm], wood (oak, thickness of 5 mm), and high density polyethylene (PE-HD, thickness of 3 mm) substrates were purchased from Rocholl GmbH, Germany. Stainless steel AISI 316 (SS, thickness of 1.95 mm) substrates were kindly supplied by Oribay Group Automotive S.L., Spain.

### 2.2 Characterization Techniques

^1^H NMR spectra were recorded on a Bruker Advance DPX 300 spectrometer at 25 °C. Deuterated dimethyl sulfoxide (DMSO-*d*_6_) was used as solvent. FT-IR spectra were obtained using an FT-IR spectrophotometer (Nicolet is20 FT-IR, Thermo Scientific Inc., USA) equipped with attenuated total reflectance (ATR) with a diamond crystal. Spectra were recorded between 4000 and 600 cm^–1^ with a spectrum resolution of 4 cm^–1^. All spectra were averaged over 16 scans. The spectra at high temperatures were obtained using an FT-IR spectrophotometer (Nicolet 6700FT-IR, Thermo Scientific Inc., USA) equipped with a specap variable temperature transmission cell. Spectra were recorded between 4000 and 400 cm^–1^ with a spectrum resolution of 4 cm^–1^, and 64 scans were signal averaged. Samples were prepared by dissolving in THF and casting on KBr windows. A differential scanning calorimeter (DSC-Q2000, TA Instruments Inc., USA) was used to analyze the thermal behavior of the samples. 6–8 mg of the samples was scanned from −70 to 120 °C at a heating rate of 20 °C min^–1^. The glass transition temperatures (*T*_g_) were taken from the inflection point in the heat capacity curve. Dynamic mechanical temperature analysis (DMTA) experiments were performed using a rectangular sample of the cross-linked materials (2 × 3.5 × 1 mm), using a Triton 2000 DMA from Triton Technology in bending mode. Tests were performed at 1 Hz, at a heating rate of 4 °C min^–1^ from −35 to 160 °C. Size exclusion chromatography (SEC) was performed in THF at 35 °C (flow rate of 1 mL min^−1^) using a Waters chromatograph equipped with three columns in series (Styragel HR1, HR2, and HR4) with increasing pore sizes (from 100 to 10^6^ Å). Toluene was used as a marker. Polystyrenes of different molar masses, ranging from 106 to 436,000 g mol^–1^, were used for the calibration. Atomic force microscopy (AFM) measurements were carried out under ambient conditions using an AFM Dimension ICON (Bruker). Topography AFM images were collected in tapping mode employing TEST-V2 tips with a resonance frequency of 320 kHz and a spring constant of 37 N m^–1^. Samples were prepared casting 0.15 mL of a 20 mg mL^–1^ solution of the polymer on glass and spin-coating at 1000 rpm for 30 s, obtaining a coating of the polymer over the glass.

#### 2.2.1 Rheological Measurements

Temperature and frequency sweep experiments were performed in a stress-controlled Anton Paar Physica MCR101 rheometer. Temperature sweep measurements were carried out from −10 to 120 °C (some experiments were finished before due to inconsistency of the data), at a frequency of 1 Hz and a strain in the linear viscoelastic range of the materials, which value depended on the studied polymer, using the 15 mm disposable parallel plate geometry. Frequency sweep experiments were carried out at a constant temperature in the linear viscoelastic regime of the materials, from 0.01 to 100 Hz (0.0628 to 628 rad s^–1^). The experiments were carried out using the 15 mm disposable parallel plate geometry. The typical terminal behavior of nonstructured polymers is characterized with eqs 1 and 2.^29,30^
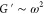
1
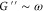
2where *G*′ is the storage modulus, *G*′’ is the loss modulus, and ω is the angular frequency in rad s^–1^.

Therefore, the linear fit of the double logarithm plots gives rise to slopes of 2 for *G*′ and 1 for *G*′’ (eqs 3 and 4).
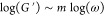
3where *G*′ is the storage modulus, ω is the angular frequency in rad s^–1^, and *m* is equal to 2.
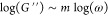
4where *G*′’ is the loss modulus, ω is the angular frequency in rad s^–1^, and *m* is equal to 1.

Linear fits of the curves at low frequencies were performed employing the data analysis tools of Origin 2020b.

#### 2.2.2 Shaping of Hot-Melt Adhesives

To prepare the hot-melt adhesive samples, ∼190 mg (rheology), ∼90 mg (probe tack), ∼250 mg (lap-shear), or ∼500 mg (SAFT and shear resistance) of the polymer was shaped in an oven between two substrates (Teflon paper sheets were used to avoid adhesion to the substrates) with spacers to control the thickness (0.6–0.8 mm). Circular samples of 15 mm diameter (rheology), around 8–10 mm diameter (tack probe), rectangular samples (12.5 × 25 mm^2^), and squares of 25 × 25 mm (625 mm^2^) (SAFT and shear resistance) were prepared. In the case of hybrid hot-melt adhesives made by adding 15 wt % D.E.R. 671, a 1 kg weight was placed over the second substrate to accelerate the shaping of the hot-melt adhesive.

#### 2.2.3 Probe Tack Tests

Probe tack measurements were carried out using a TA.HDPlus texture analyzer (Texture Technologies, Hamilton, MA, USA) under controlled temperature in an oven. A 5 mm stainless steel cylinder probe was moved downward at a speed of 0.1 mm s^-1^ until it was brought into contact to the adhesive surface. Immediately after the contact (10 s of dwelling time, with a compressive force of 1 N), the crosshead was allowed to move upward at a speed of 300 mm min^–1^ until the probe was completely separated from the adhesive. Tests were performed on 3 samples for each formulation to determine the average lap-shear strength, and the standard deviation was used as the error.

#### 2.2.4 Lap-Shear Tests

The adhesion properties of the PHUs were evaluated at room temperature using an Instron 5569 and applying a parallel force to the adhesive bond with a displacement rate of 50 mm min^–1^. Corresponding substrates with dimensions of 100 mm × 25 mm × (thickness of each substrate) mm were bonded with an adhesive contact area of 312.5 mm^2^ (25 mm × 12.5 mm). The gripping length on both sides of the test specimens was 25 mm. Tests were performed on 5 samples for each formulation to determine the average lap-shear strength, and the standard deviation was used as the error. The nature of adhesion failure was also recorded based on visual inspection of the sample following the test.

Lap-shear test specimens were prepared as follows. The surfaces of the substrates were cleaned following the procedure described elsewhere.^31^ Hot-melt adhesive samples were applied after shaping onto one of the pairs of the cleaned substrates and were introduced into an oven (Memmert Vacuum Drying Oven VO200, Thermo Scientific Inc., USA) at 80 or 100 °C for 5 min, allowing the softening of the polymer. Afterward, both substrates were put together and placed again into the oven for 5 min at the corresponding temperature (80 or 100 °C). The adhesive joints were stored at ambient temperature for 24 h prior to testing.

For hybrid hot-melt adhesives, polymers were put together with the substrates and directly placed in the oven at 120 °C for 10 min with 1 kg over the glue line to improve the wetting of the adherends.

After finishing the lap-shear test, the thermoreversible adhesion of the adhesives was tested, to prove the efficiency of the material for repeated use. Each pair of substrates was stuck again and was placed into the oven under the same conditions of the first application. Following the same procedure, lap-shear measurements were repeated.

Subsequently, a second rebonding was done. In this case, adhesives were heated and removed from the substrates manually. The test specimens were prepared, and lap-shear measurements were carried out following the same procedure, employing the recycled material for shaping the adhesives.

#### 2.2.5 Shear Adhesion Failure Temperature (SAFT) Tests

Shear tests were performed on stainless steel panels using SAFT equipment. Specimens were prepared following the procedure described for lap-shear tests. Specimens were conditioned at 298 K with 50% of R.H. for 1 day. A mass of 1000 g was hung to each panel, and they were placed into an oven. The temperature was increased from 30 to 217 °C at a heating rate of 1 °C min^–1^. The temperature of failure was reported together with the nature of adhesive failure. Tests were performed on 4 samples for each formulation to determine the average lap-shear strength, and the standard deviation was used as the error.

#### 2.2.6 Shear Resistance Tests

Shear resistance tests were performed following the same procedure and using the same equipment as the one described for SAFT experiments. Instead of applying a heating rate, temperature was set at 30 °C and the time of failure was recorded. The nature of adhesion failure was also recorded based on visual inspection of the sample following the test.

## 3 Results and Discussion

### 3.1 Preparation of Model Hot-Melt PHU adhesives

**Scheme 1:**
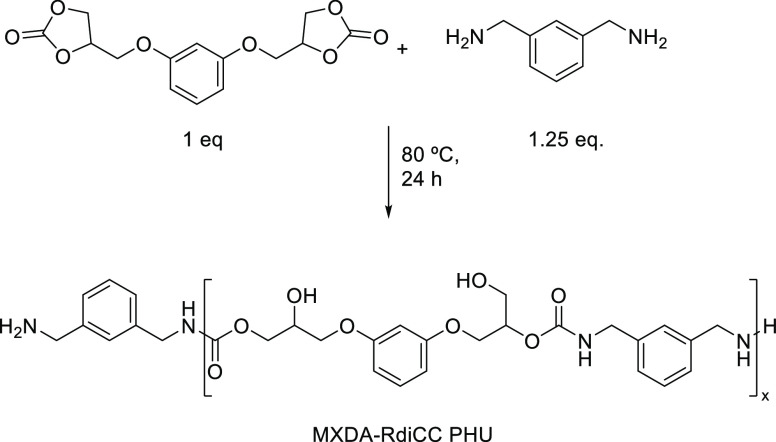
Synthesis of PHU Homopolymers from RdiCC and MXDA

In our quest to hot-melt adhesives, a PHU made of a rigid diamine such as *m*-xylylenediamine (MXDA) combined with resorcinol dicyclic carbonate (RdiCC) was initially synthesized at 80 °C for 24 h (Scheme 1).

The completion of the reaction was followed by FTIR-ATR and ^1^H NMR, which are described in the Supporting Information (Figures S1 and S2, respectively).

**Scheme 2:**
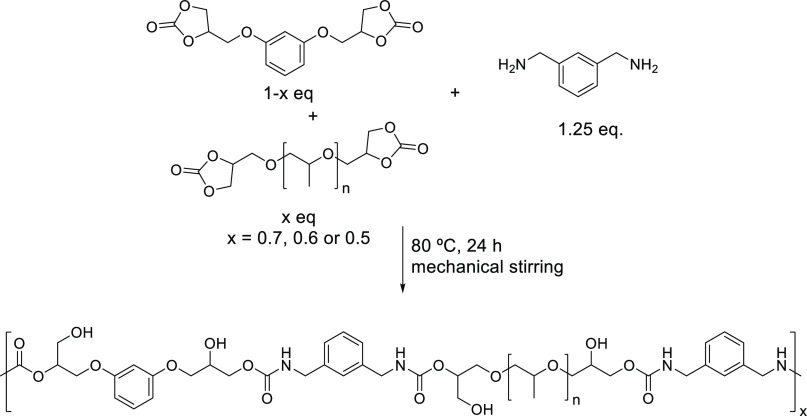
Preparation of PHU Copolymers from PPGdiCC, RdiCC, and MXDA

Thermal characterization of the MXDA-RdiCC PHU was performed by differential scanning calorimetry (Figure S3). The glass transition temperature (*T*_g_) was calculated from the second heating scan and estimated to be 51 °C, confirming its solid state at ambient conditions. As a result, this material is too stiff at room temperature to be used as hot-melt adhesives. We have recently reported the importance of the balance between hard and soft segments for designing adhesives with optimal properties.^31,32^ Therefore, polypropylene glycol dicyclic carbonate (PPGdiCC) was introduced in the RdiCC/MXDA formulation to bring softness to the PHU HMAs,

In order to study the effect of the soft segment on the adhesive properties, three formulations varying the PPGdiCC:RdiCC molar ratios (70/30, 60/40, and 50/50) were used and were reacted with MXDA at 80 °C for 24 h (samples named 70/30-MXDA, 60/40-MXDA, and 50/50-MXDA, Scheme 2). For comparative purposes, a homopolymer based on pure PPGdiCC was also prepared. The copolymerizations were also monitored using FTIR-ATR and ^1^ H NMR spectroscopy. As expected the characteristic bands of the carbonate group both in the FTIR (1780–1790 cm^–1^) (Figure S4) and in the ^1^H NMR (5.02, 4.62–4.53, and 4.23–4.13 ppm) disappeared and new bands attributed to the formation of the urethane carbonyl at 1696 cm^–1^ and at 7.6–7.8 ppm (-NH-C(O)O) and at 4.15 ppm (-CH_2_NH-) were observed, which is in agreement with the expected ring-opening of the cyclic carbonates (Figures S5, S6, S7, and S8). In Figure S3 the DSC curves of all the copolymers and the homopolymer based on PPGdiCC carbonate are shown. As expected, the homopolymers based on PPGdiCC exhibited the lowest *T*_g_ (−11 °C) while the rest of the materials have intermediate *T*_g_ values ranging from 0 to 9 °C which are more appropriate for low temperature hot-melt adhesive applications.^33^

**Figure 1:**
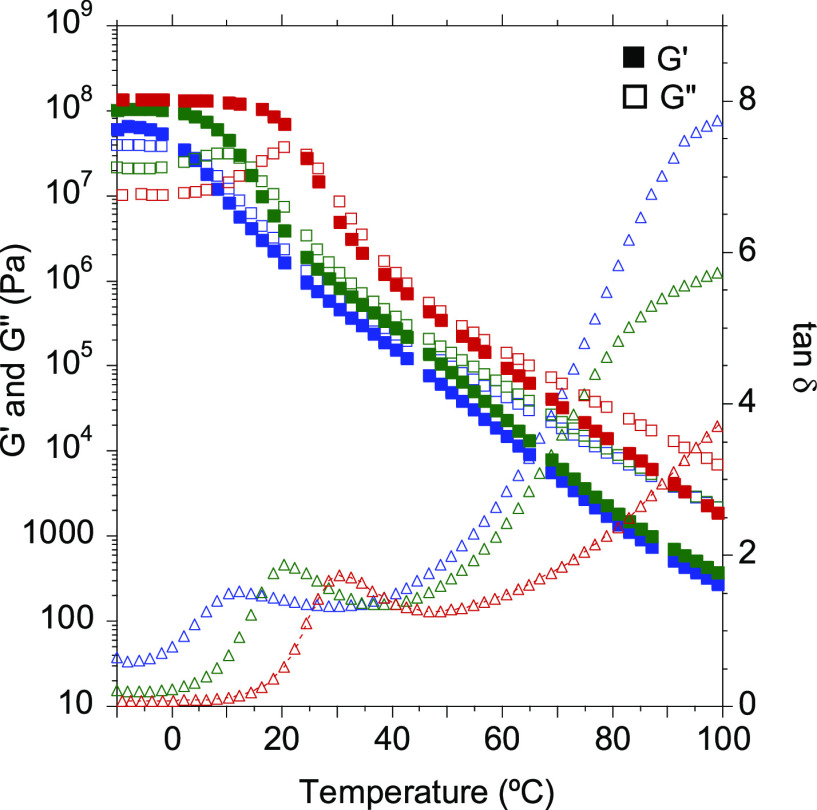
Storage (*G*′) and loss (*G*′’) moduli and tan δ values for 70/30-MXDA (blue), 60/40-MXDA (green), and 50/50-MXDA (red) compositions between −10 and 100 °C.

Then, dynamic rheological measurements were performed to get a better understanding of the viscoelastic response of the PHUs. Both temperature sweep as well as frequency sweep experiments were carried out to elucidate the behavior of the adhesives as hot-melts. Figure 1 shows the evolution with temperature of the storage (*G*′) and loss (*G*′’) moduli and the tan δ for the different copolymer compositions.

As observed in the DSC thermograms, the *T*_g_ decreased as the PPGdiCC molar ratio was increased because the chain mobility was enhanced. Thus, for the 70/30-MXDA composition richer in flexible PPGdiCC, a *T*_g_ value of 10.3 °C was measured. This value increased up to 30.5 °C when an equimolar amount of both dicyclic carbonates was employed (50/50-MXDA). Moreover, the 50/50-MXDA formulation showed a slower decay of the moduli after the *T*_g_, probably due to the greater density of hydrogen bonds. Shorter lengths between urethane groups gives rise to a higher density of hydrogen bonds.^24^ In addition, this decay was not as pronounced as it should be for polymers that do not present any supramolecular interactions.

**Figure 2:**
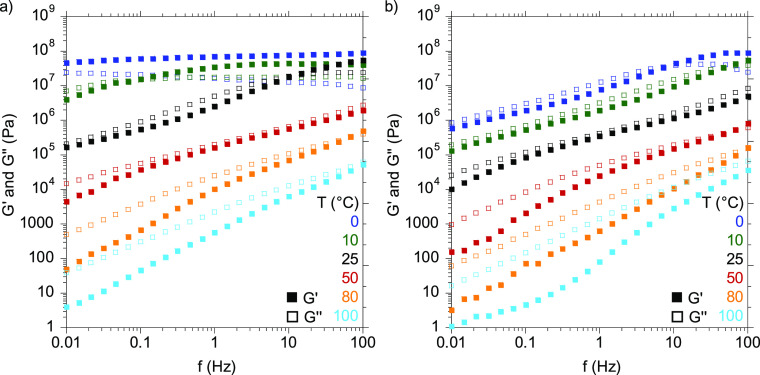
(a) *G*′ and *G*′′ values between 0.01 and 100 Hz at different temperatures of (a) 50/50-MXDA and (b) 70/30-MXDA.

In order to confirm the presence or absence of these interactions within the formulations, the adhesives were characterized by frequency sweep measurements at 0, 10, 25, 50, 80, and 100 °C (Figure 2).

Frequency sweep measurements showed that below the *T*_g_, 50/50-MXDA presented a solid-like behavior, and at 0 °C, *G*′ remained above *G*′′ over the whole range of frequency. With the increase of temperature, the transition crossover point from solid-like to liquid-like behavior (*G*′ = *G*′′) moved to higher frequencies. This is due to the greater mobility of the chains at higher temperatures, and therefore, the material exhibits a liquid-like behavior at shorter times. On the other hand, the more liquid-like behavior of the 70/30-MXDA was confirmed, as this transition occurred at higher frequency when compared with 50/50-MXDA at the same temperatures. Above the *T*_g_, both compositions presented a liquid-like behavior at low frequencies, where *G*′′ was always higher than *G*′. For nonstructured materials this terminal zone is characterized by slopes of −2 and −1 for *G*′ and *G*′′, respectively, in the double logarithmic representation and corresponds to a flow regime.^29,30,34,35^ However, in the case of the PHU adhesives, the slope for the curves differed from these values, even at 100 °C (Figure S9). This suggests the formation of structured polymer networks through hydrogen bonds between the poly(hydroxy urethane)s chains, which hinder the flowing of the material. Moreover, this process is fully reversible as when carrying a second frequency sweep, *G*′ as well *G*′′ were practically equal to the first measurement, supporting the presence of reversible hydrogen bonds (Figure S10).

**Figure 3:**
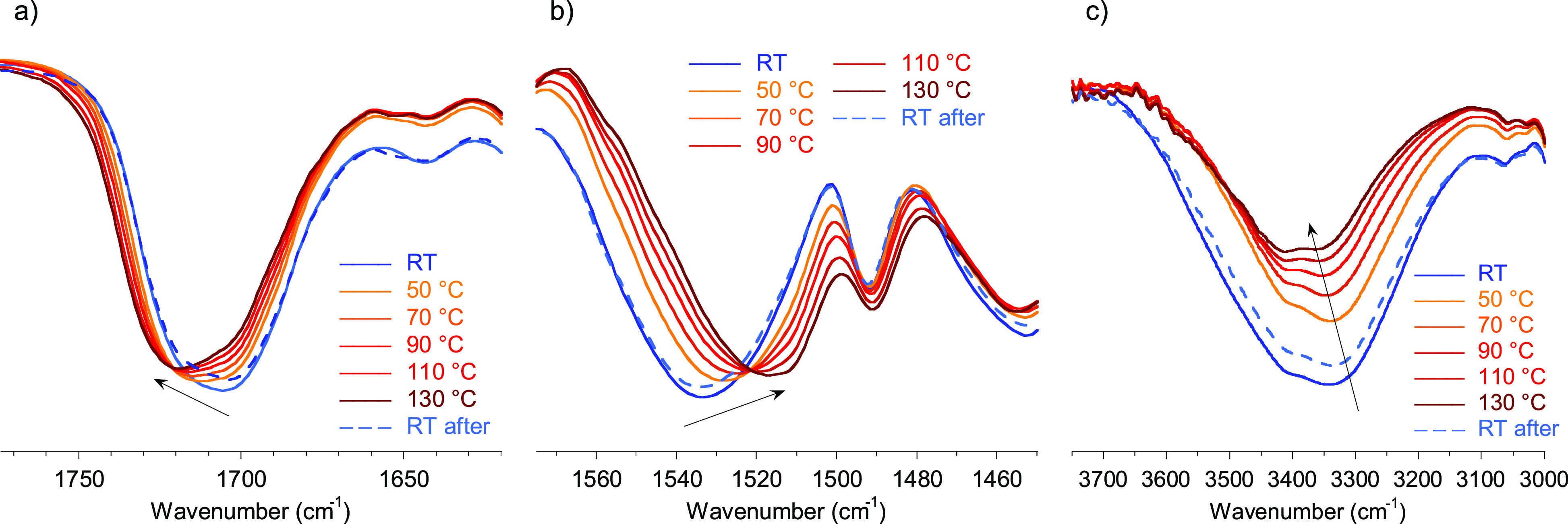
Variable temperature FTIR spectra in the region of the (a) C=O stretching band, (b) N–H bending band, and (c) O–H stretching band of the 50/50-MXDA composition.

As additional proof of hydrogen bond formation, FTIR spectra were recorded at different temperatures (Figure 3). It is well established that hydrogen bonding and debonding can be followed through the displacement of the urethane C=O stretching vibration, N–H bending vibration, and O–H stretching vibration of the hydroxyl groups.^35−39^ The C=O stretching band region of the infrared spectra is shown in Figure 3a. At room temperature, signals at 1720 and 1705 cm^–1^, which are related to free and hydrogen bonded carbonyls, respectively, can be observed. The relative absorbance of the hydrogen bonded band is higher, indicating that the majority of the carbonyl groups are taking part in the formation of hydrogen bonds (that can happen with the N–H and OH).^36−38^ When the temperature was increased, the absorbance of the peak at 1720 cm^–1^, related to the free carbonyl groups, increased. However, the remaining shoulder at 1705 cm^–1^ shows that hydrogen bonds were still present, even at 130 °C. Following the same trend, the N–H bending band red-shifted from 1534 to 1517 cm^–1^ (Figure 3b) when temperature increased due to the disappearance of the anchoring restriction from hydrogen bonding.^39^ Finally, the intensity of the maximum OH band at 3344 cm^–1^ (related to hydrogen bonded hydroxyl groups) blue-shifts with temperature (Figure 3c). Because of the weakening of the hydrogen bonds with temperature, a greater energy was necessary to excite the O–H bonds.^35^ Interestingly, when the sample was cooled down, these signals returned to the initial wavenumber values, demonstrating the reversibility of the hydrogen bonds.

Characterization of the 50/50-MXDA composition by AFM showed that the copolymer presented some phase separation at the nanoscale, confirming that the rigid diamine can partly prevent hydrogen bonding formation, reducing the phase mixing of hard and soft domains (Figure S11).

In sum, combination of both soft and hard segment cyclic carbonates allowed preparation of good PHU candidates for hot-melt adhesives. Incorporation of greater amounts of hard segment hindered the transition to liquid-like behavior material. Rheological measurements confirmed the formation of structured materials through hydrogen bonding, which was further confirmed by FTIR-ATR spectra.

### 3.2 Influence of the Monomer Structure on the Rheological Behavior of PHUs

#### 3.2.1 Copolymers Based on Aliphatic Dicyclic Carbonates

**Scheme 3:**
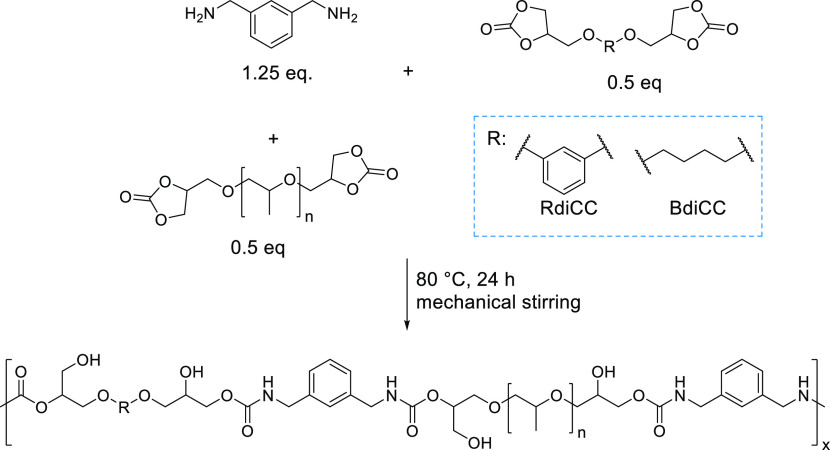
Synthesis of the Copolymers Based on PPGdiCC, BdiCC, and MXDA

In order to elucidate if the phase separation in PHUs was due to the aromaticity of the employed cyclic carbonate, resorcinol dicyclic carbonate was substituted for an aliphatic one, 1,4-butanediol dicyclic carbonate (BdiCC). The synthesis was performed following the procedure employed for the preparation of the previous copolymers (Scheme 3). FTIR and ^1^H NMR characterization of the PHU obtained is reported in Figures S12 and S13, respectively.

##### 3.2.1.1 Rheological Behavior of 50/50BdiCC-MXDA

**Figure 4:**
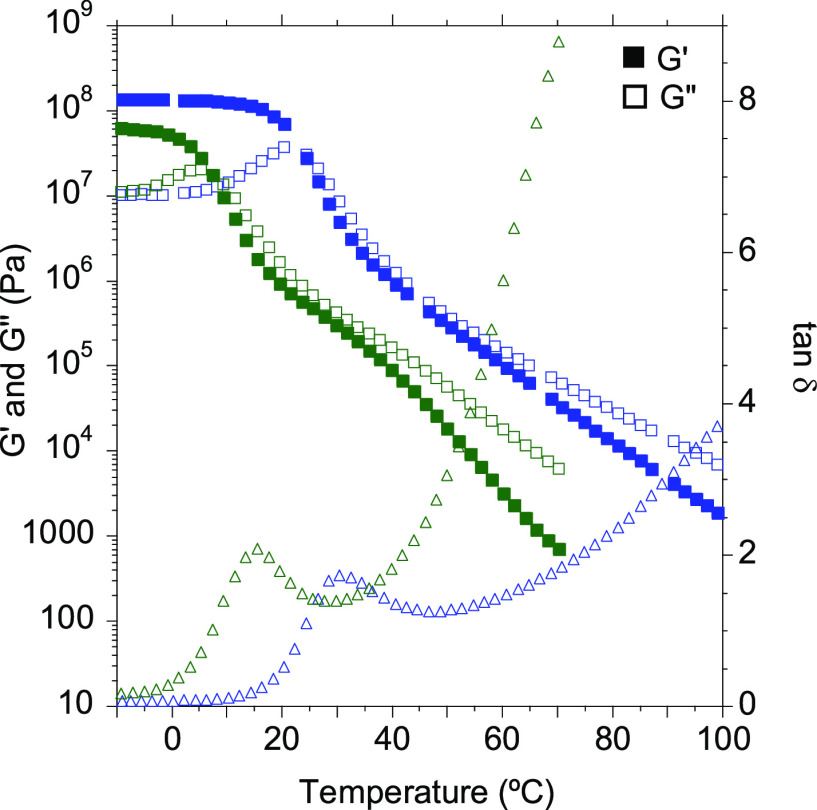
*G*′, *G*′′, and tan δ values of 50/50-MXDA (blue) and 50/50BdiCC-MXDA (green). 50/50-MXDA was represented again for easier comparison of the data. The curve of 50/50BdiCC-MXDA was stopped at lower frequencies due to the high signal-to-noise ratio caused by the low moduli values.

Aiming to elucidate the viscoelastic behavior of the copolymer based on aliphatic dicyclic carbonates, temperature and frequency sweep measurements were carried out. In a first step, the storage (*G*′) and loss (*G*′′) moduli as well as loss tangent (tan δ) were evaluated as a function of temperature (Figure 4). As expected, the substitution of the aromatic RdiCC for the aliphatic BdiCC gave rise to less rigid materials with a 2-fold decrease of the glass transition temperature from 30.5 to 15.5 °C (Table S2). The decay of the moduli was also faster, showing a greater liquid-like behavior when BdiCC was employed. Frequency sweep measurements corroborated the faster decay of the moduli over the whole range of temperatures and a higher dependency of these moduli with frequency (Figure S14). Nevertheless, although the moduli values were quite different, HMAs could be obtained with different properties when aliphatic cyclic carbonates were employed to prepare the PHU.

#### 3.2.2 Tuning PHU Hot-Melt Adhesives by Changing the Diamine Structure

##### 3.2.2.1 Synthesis and Characterization of Copolymers Based on Different Diamines

**Scheme 4:**
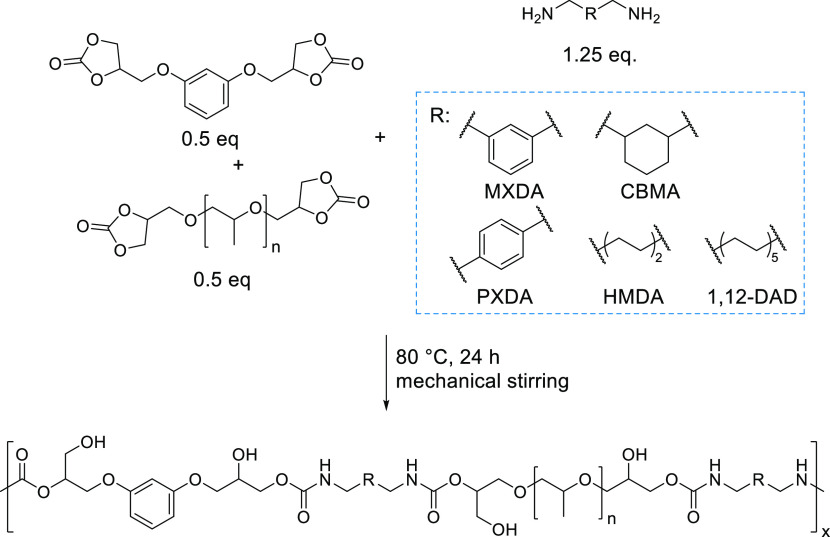
Preparation of PHU Copolymer Compositions Reacting a 50/50 Molar Ratio of PPGdiCC/RdiCC with the Corresponding Diamine, i.e. *m*-Xylylenediamine (MXDA), *p*-Xylylenediamine (PXDA), 1,3-Cyclohexanebis(methylamine) (CBMA), Hexamethylenediamine (HMDA), and 1,12-Diaminododecane (1,12-DAD)

After confirming that HMA could be obtained with both aromatic and aliphatic dicyclic carbonates, we investigated the impact of the diamine on the HMA behavior. To do so, new copolymers based on a 50/50 molar ratio of the dicyclic carbonates varying the diamine were prepared following the procedure of the MXDA-based copolymers (Scheme 4). The formation of the PHU was confirmed by FTIR-ATR (Figure S15) and ^1^H NMR (Figures S16 and Figure S17). Surprisingly, the samples prepared using HMDA and 1,12-DAD aliphatic diamines were not soluble in common solvents such as THF, alcohols (MeOH, EtOH, or IPOH), DMF, DMAc, and DMSO, which easily dissolved the synthesized aromatic-based PHUs.

**Figure 5:**
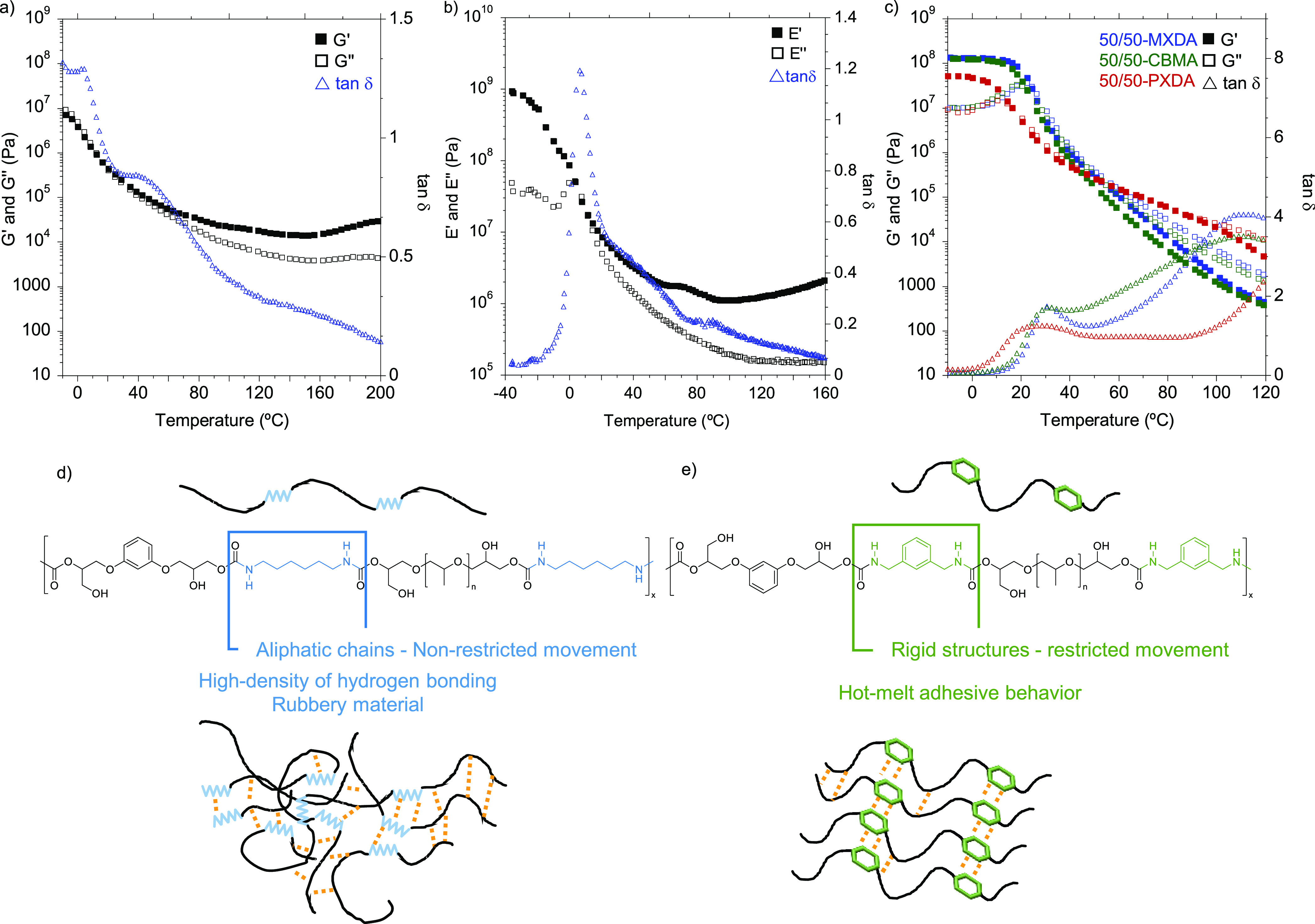
(a) *G*′, *G*′′, and tan δ values of 50/50-HMDA from temperature sweep measurements. (b) *E*′, *E*′′, and tan δ values of 50/50-DAD from DMTA analysis. (This composition was studied through DMTA analysis due to the easier sample preparation.) (c) *G*′, *G*′′, and tan δ values of 50/50-MXDA (blue), 50/50-CBMA (green), and 50/50-PXDA (red). 50/50-MXDA was represented again for easier comparison of the data. Schematic representation of the hydrogen bonding (in orange) hypothesis for (d) nonrestricted movement aliphatic diamine-based PHUs and (e) restricted movement rigid ring-based diamines.

Temperature sweep experiments were carried out to address the viscoelastic behavior of the materials (Figure 5). When using the two aliphatic diamines, HMDA as well as 1,12-DAD, we did not observe any HMA behavior as both materials exhibited larger values of the storage (*E*′ or *G*′) than the loss modulus (*E*′′ or *G*′′) over the whole range of temperatures, which is typical for cross-linked materials (Figure 5a and b). Moreover, when frequency sweeps were carried out, *E*′ and *E*′′ exhibited a low dependence on frequency, demonstrating the permanent elastic behavior of the material (Figure S18a). These samples also presented high gel content after Soxhlet extractions in refluxing THF (Table S2). It is thought that in this case the interactions between hydroxyl groups and urethanes groups are stronger than in the case of aromatic diamines due to the greater mobility and lower steric hindrance of the aliphatic chains, which decrease the likelihood of the material to flow (Figure 5d). This result shows that rigid diamines favored selective formation of hydrogen bonding ideal for preparing HMAs (Figure 5e).

In order to evaluate if this selective hydrogen bonding could be derived from π–π interatctions, both aromatic and cycloaliphatic diamines were investigated, including 50/50-CBMA and 50/50-PXDA (Figure 5c). These two diamines showed similar behavior to 50/50-MXDA, and above the glass transition temperatures both formulations suffered from a progressive decrease of the moduli. It has to be noted that this decrease of the moduli was a bit more pronounced when cycloaliphatic diamine, CBMA, was employed, whereas when PXDA was used, the moduli remained higher with *G*′ ≈ *G*′′, indicating a stronger interaction of the chains. Frequency sweep measurements were carried out for further analysis of the rheological behavior of the adhesives (Figure S19).

Frequency sweep measurements at 80 °C of 50/50-CBMA exhibited a similar decay of the moduli to the 50/50-MXDA sample. Thus, possible π–π contributions were discarded to be the dominant cause of the interactions between PHU chains. On the other hand, 50/50-PXDA showed a crossover between *G*′ and *G*′′ (Figure S19d, red spots). At high frequencies, this material presented a solid-like behavior (*G*′ > *G*′′), while at low frequencies, the adhesive behaved like a liquid (*G*′′ > *G*′). In the whole range of frequencies at 80 °C, adhesives based on PXDA exhibited larger storage as well as loss moduli than MXDA-based materials, demonstrating a key role of the aromatic ring substitution in hydrogen bond interactions. We can conclude that this selective hydrogen bonding is more related to the conformational effect of the diamine that we use. Thus, rigid diamines limit to some extent hydrogen bonding in PHUs, favoring the formation of HMAs.

#### 3.2.3 Adhesive Properties of PHU Copolymers

The adhesive properties of all PHU formulations were evaluated through probe tack, lap-shear, shear adhesion failure temperature (SAFT), and static shear resistance experiments. The influence of the soft to hard segment ratio in samples based on MXDA having 50/50, 60/40, and 70/30 of PPGdiCC and RdiCC, respectively, was investigated. Subsequently, the impact of the nature of the dicyclic carbonate and the diamine was further explored.

##### 3.2.3.1 Dynamic Adhesive Properties

**Figure 6:**
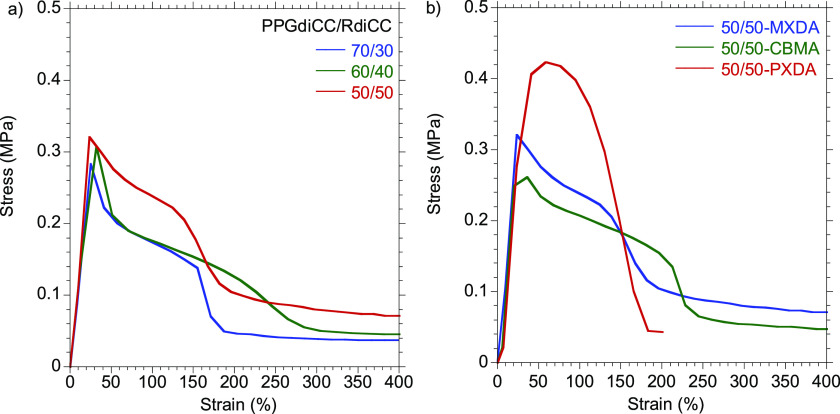
Probe tack stress–strain curves performed at 100 °C for (a) 70/30-, 60/40-, and 50/50-MXDA compositions and (b) 50/50-MXDA, -CBMA, and -PXDA compositions.

First, the tackiness of the hot-melt PHUs was probed by tack measurements at two temperatures, 80 and 100 °C. The tack force was evaluated by the maximum stress and capacity of PHU adhesives for wetting the surface by the evolution of the curves and fibrillation of the adhesive. Tests were performed using a 5 mm stainless steel cylinder, allowing 10 s of contact between the probe and the adhesive at the corresponding temperature, employing a debonding speed of 300 mm s^−1^. Stress–strain curves of the compositions at 80 and 100 °C are depicted in Figure S20 and Figure 6a, respectively.

It was found that regardless of the rigid/soft ratio, the formulations based on MXDA manifested a similar behavior, exhibiting similar maximum stresses, although at 100 °C the value was lower due to the higher liquid-like behavior of the adhesives at this temperature. At 80 °C, 50/50-MXDA presented a sharp decrease after the maximum stress and an adhesive failure after the test. The cohesion of the material was higher than adhesion forces, hindering fibrillation of the adhesive. Therefore, a higher temperature would favor application of the adhesive and the wetting of the adherends. On the contrary, 70/30- and 60/40-MXDA showed a slight stabilization of the stress (plateau), which was indicative of the creation of a fibrillating structure. The latter presented a greater capacity for fibrillation since a higher plateau was achieved. At 100 °C, all compositions exhibited fibrillation, i.e. capacity for wetting the surface. The 50/50-MXDA formulation was able to retain cohesive properties to a greater extent, which correlated with the higher *G*′ values at 100 °C in the rheological experiments.

On the other hand, the influence of the diamine structure on the tackiness of the adhesives was further examined. Probe tack stress–strain curves of MXDA-, CBMA-, and PXDA-based PHUs are shown in Figure 6b. According to rheology frequency sweeps, the 50/50-PXDA composition showed a greater cohesiveness, and no formation of the plateau was observed. Due to these higher cohesive forces, adhesive failure was recorded while no residue remained on the stainless steel cylinder probe. As the formulation did not present liquid-like behavior at this temperature, a further experiment was performed at 120 °C (Figure S21). The maximum stress of the 50/50-PXDA was slightly lower, but the plateau was extended, exhibiting higher capacity to wet the surface. Nonetheless, adhesive failure was still recorded, and no fibrillation was observed, showing similar solid-like behavior at these test conditions.

**Figure 7:**
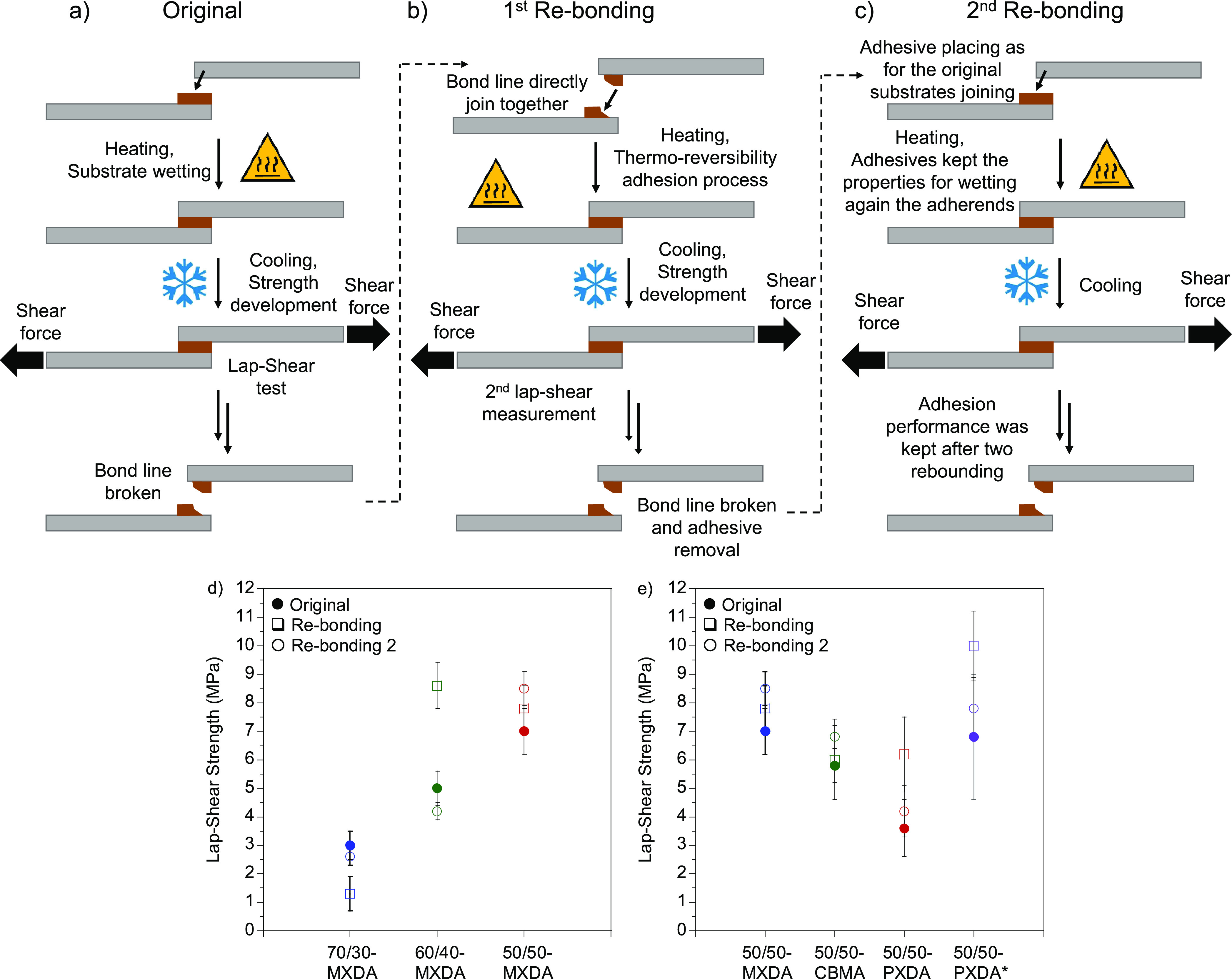
Schematic representation of the process for bonding stainless steel adherends for (a) the first time, (b) a first rebonding after breaking the bond line, and (c) a second rebonding after breaking two times the joint. Lap-shear strength values when samples were applied onto the substrates at 100 °C of the (d) 70/30-, 60/40-, and 50/50-MXDA formulations and (e) 50/50-MXDA-, -CBMA-, and -PXDA-based compositions. 50/50-PXDA* (purple) was applied at 120 °C. The lap-shear strength values are the average of five test specimens, and the error bar represents the standard deviation.

After assessing the tackiness of the adhesives, the lap-shear strength was determined. Lap-shear specimens of samples with varying soft to hard segment ratio were prepared as shown in Figure 7a, by bonding the stainless steel substrates at 80 and 100 °C (Figure S22 and Figure 7d, respectively).

When the materials were applied at 80 °C, the 70/30-MXDA was characterized by a low lap-shear strength, i.e., 1.9 ± 0.6 MPa, while the 60/40-MXDA and 50/50-MXDA showed similar and better performance (Figure S22) with a lap-shear adhesion of 2.5–3 MPa. On the other hand, raising the temperature up to 100 °C enhanced the performance of the adhesives, especially in the case of 50/50-MXDA (Figure 7d) to 7 MPa.

This observation correlated with probe tack measurements, where the 50/50-MXDA exhibited a greater liquid-like behavior and, therefore, improved wetting of the surface of the adherends. Adhesive failure was predominant for all the compositions regardless of the application temperature, indicating greater cohesive forces of the polymer than adhesion between interfaces (Figures S23a–c and S24a–c).

After evaluating the impact of the soft to hard segment ratio, the effect of BdiCC on the adhesion properties was investigated, replacing RdiCC by this monomer. According to the rheological behavior showed by this composition, the adhesion properties (2 ± 0.4 MPa) were far below the adhesion performance of 50/50-MXDA in all the measurements (Figure S25 and Table S3). Adhesive failure of the PHU was recorded showing greater cohesive forces than interaction with the adherend (Figure S26a).

Finally, the influence of the diamine structure on lap-shear experiments was addressed. Based on rheology and better lap-shear results of the initial study, hot-melt adhesives were applied at 100 °C. The MXDA-based formulation presented slightly better lap-shear strength than the CBMA-based composition (7 MPa vs 5.8 MPa), while the difference between the 50/50-PXDA when it was applied at 100 and 120 °C demonstrated the necessity of good wetting of the surface to obtain optimal adhesive performance (Figure 7e) as attested by a nearly 2-fold increase of the lap-shear adhesion values.

Adhesive failure after lap-shear tests was predominantly observed for the compositions (Figure S27), demonstrating the greater cohesive forces of the polymers than their adhesion affinity for the adherend.

In brief, dynamic adhesive properties were dependent on the balance between soft and hard segments, increasing the adhesive performance when a greater amount of RdiCC hard segment was added into the formulation. On the other hand, substitution of the aromatic cyclic carbonate by aliphatic BdiCC was detrimental for this adhesive performance. Finally, a similar performance was observed when formulations were prepared with different cyclic diamines, with the selection of an adequate application temperature being crucial to obtain good results.

##### 3.2.3.2 Thermoreversibility

In hot-melt technologies, in which the adhesive flows at elevated temperature and solidifies when the temperature is decreased below their *T*_g_ or *T*_m_, the reversibility of the adhesive should be demonstrated to show the potential of a given HMA to adhere several times. Thermoreversible adhesion of the polymers was addressed by rebonding adhesives two times (Rebonding and Rebonding 2, Figure 7b and c, respectively) after breaking the bond line. In a first rebonding, substrates were joined together directly and put into the oven at the corresponding temperature allowing breaking of the interchain interactions and ensuring some chain mobility. After cooling down the material below *T*_g_ of HMA, substrates were bonded again. In a second rebonding, adhesives were removed totally from the substrates and applied to new specimens. As a general trend, similar or better lap-shear strength values confirmed the efficiency of the materials for a second or third use. Adhesive failure after the test was almost exclusively observed, with the exception of the 50/50BdiCC-MXDA composition, which presented cohesive failure (Figures S23d–i, S24d–i, S26b and c, and S27d–i).

##### 3.2.3.3 Static Adhesive Properties

Additional adhesive characterization was performed through shear adhesion failure temperature (SAFT) and shear resistance measurements. The first measurement is intended to determine the temperature at which specimens suffer from delamination under static load in shear (service temperature), whereas the shear resistance provides the resistance of the materials to creep under static load in shear under a constant temperature.

Higher service temperatures as well as creep resistance values were achieved when the proportion of the hard segment, RdiCC, was increased, in agreement with *T*_g_ values and the more solid-like behavior manifested in the rheological measurements (Table 1). On the other hand, better results were obtained when the adhesives were applied onto the substrates at 100 °C, especially for 50/50-MXDA, bringing to light the necessity of higher temperature for the good wetting of the substrate. After performing these tests, cohesive failure (Figure S28) was observed regardless of the composition or temperatures of application, confirming the liquid-like behavior obtained at low frequencies during frequency sweep measurements (Figure 2).

**Table 1:** Shear Adhesion Failure Temperature (SAFT) and Shear Resistance Values for PHU Hot-Melt Adhesives

		SAFT (°C)		Shear Resistance (min)	
Entry	Code	Applied at 80 °C	Applied at 100 °C	Applied at 80 °C	Applied at 100 °C
1	70/30-MXDA	42 ± 1	43 ± 1	11 ± 1	31 ± 1
2	60/40-MXDA	42 ± 1	47 ± 1	86 ± 1	100 ± 7
3	50/50-MXDA	52 ± 2	59 ± 1	196 ± 32	371 ± 18
4	50/50-PXDA	a	63 ± 1	a	1941 ± 47
5	50/50-CBMA	a	50 ± 2	a	160 ± 47

a Adhesives were applied only at 100 °C.

Afterward, SAFT and shear resistance measurements for the adhesives based on 50/50 cyclic carbonate molar ratios and different diamines were performed (Table 1, entries 3, 4, and 5). Aromatic diamine-based formulations (50/50-MXDA and -PXDA) exhibited greater SAFT as well as shear resistance compared to the cycloaliphatic diamine formulation (50/50-CBMA), corroborating the more solid-like behavior, with its lower tendency to flow, as shown in the frequency sweep measurements (Figure S19). When the aromatic diamine was *para* substituted, the moduli exhibited lower frequency dependence and, therefore, greater resistance to flow, which agreed with the 5-fold increase in performance in shear resistance experiments (Table 1). Cohesive failure was observed after all tests, confirming the liquid-like behavior of the compositions at low frequencies regardless of the diamine (Figure S29).

Nevertheless, it should be pointed out that one of the characteristics of conventional PUs is to provide adhesion at high temperatures.^40^ This fact is usually related to the ability to retain structural integrity even at high temperatures due to the presence of strong hydrogen bonding when high molar mass polyurethanes are used. One of the main drawbacks of PHUs is that they give rise to lower molar masses than conventional PUs due to the strong hydrogen bonds that are formed between carbonyl groups, the NH of the carbamate group, and the hydroxyl group formed during ring-opening of the cyclic carbonates^26^ and also due to the reversibility of the cyclic carbonate aminolysis reaction.^25^ This often limits their use where high molar mass TPUs are demanded. Size exclusion chromatography (SEC) was employed for the characterization of the average molar masses of the copolymers (Table S2). Although most of the compositions were prepared under stoichiometric imbalanced conditions, which according to Carother’s equation avoids obtaining high molar masses, these as well as the stoichiometric balanced composition presented very similar weight-average molar masses and polydispersities, in the typical values for step-growth polyaddition of dicyclic carbonates with diamines, which are in the range of 5–10 kg mol^–1^. These low molar masses might be insufficient to have an acceptable level of adhesion at elevated temperatures.

In a nutshell, the total percentage of RdiCC (hard segment), the selected diamine, as well as the temperature of application had a critical influence on the static adhesive properties of PHU hot-melt adhesives. Incorporation of *para*-substituted diamine, PXDA, the addition of a greater amount of RdiCC, and the higher application temperature resulted in adhesives with greater service temperature and creep resistance.

##### 3.2.3.4 Adhesion Performance on Different Substrates

**Figure 8:**
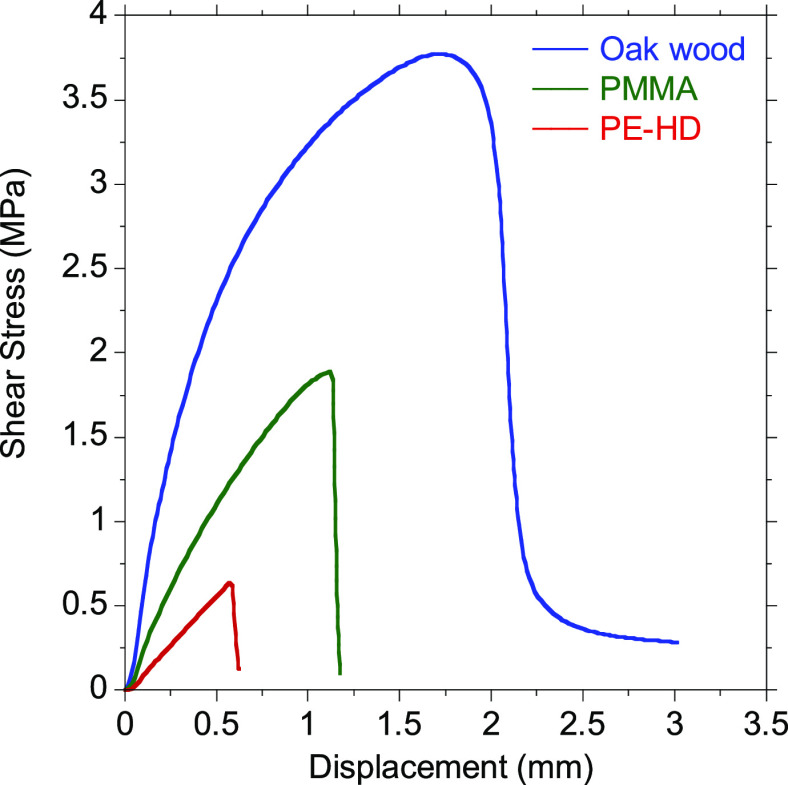
Comparison of shear stress–displacement curves of the 50/50-MXDA composition applied on oak wood, poly(methyl methacrylate) (PMMA), and high density polyethylene (PE-HD) substrates.

The ability of the hot-melt adhesives for adhering different substrates was addressed by performing lap-shear strength measurements on oak wood, PE-HD, and PMMA. These tests were performed using the 50/50-MXDA formulation. As can be seen in Figure 8, low lap-shear strength values were measured when polymeric substrates were glued.

Nevertheless, PMMA showed stronger interactions with the adhesive than PE-HD, due to its greater surface energy. As the surface energy becomes higher, it is easier to wet the substrate, and therefore, stronger attractive forces were created between the adhesive and the substrate. In addition, PMMA can establish hydrogen bonding interactions with the adhesive, while PE-HD cannot. For all polymeric substrates, adhesive failure was observed indicating a low affinity between adherend and adhesive (Figure S30). In contrast, when a polar substrate such as oak wood was bonded, higher lap-shear strength values were obtained. This demonstrates that the adhesives had a higher interaction with polar substrates, as the hydroxyl groups of the polymer chain had stronger interaction with these surfaces.

### 3.3 Hybrid PHUs

**Scheme 5:**
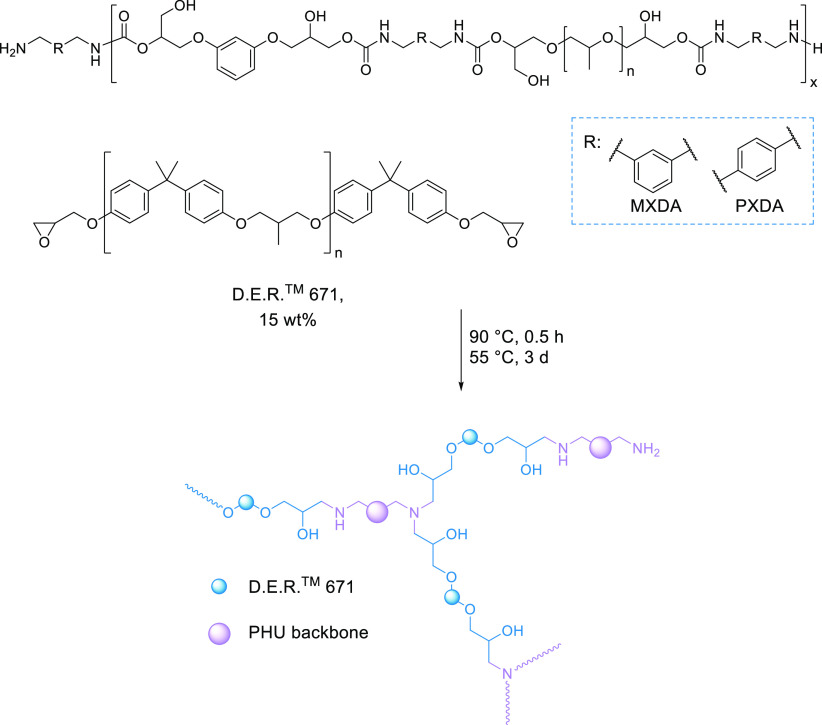
Preparation of Enhanced PHU Hot-Melt Adhesives

As discussed above, hot-melt adhesives are usually composed of more than just one component. Usually commercial HMAs also contain some additional resins (e.g., epoxy resin) in the formulation, which are used to enhance the mechanical properties. In these hybrid systems, when the resin and the polymer react, they can create a cross-linked structure that presents stronger mechanical properties than the polymer itself.^41^ Therefore, in an attempt to endow the PHU hot-melt adhesives with better static adhesive properties, i.e., SAFT, an epoxy resin was incorporated to the 50/50-MXDA and 50/50-PXDA compositions. The incorporation of additional chemistry to the PHU formulations, in this case, epoxy resins, has been demonstrated to improve the performance of PHU materials.^42−46^ The excess of diamine employed during the PHU synthesis allowed the further reaction with the epoxy resin. It has been reported that primary amine can react twice (two active hydrogens) with an epoxy ring, while already reacted amines with cyclic carbonates are not able to react with the oxirane.^46^ A possible final structure of the adhesive is shown in Scheme 5.

The PHU polymers, 50/50-MXDA and 50/50-PXDA, were heated up to 90 °C and mixed with 15 wt % solid epoxy resin, D.E.R. 671, for 20 min. Materials were allowed to react at 55 °C for 3 days to ensure the total reaction of the diamines and epoxy groups.

Materials were first characterized by FTIR-ATR showing that the PHU structure was maintained (Figure S31).

**Figure 9:**
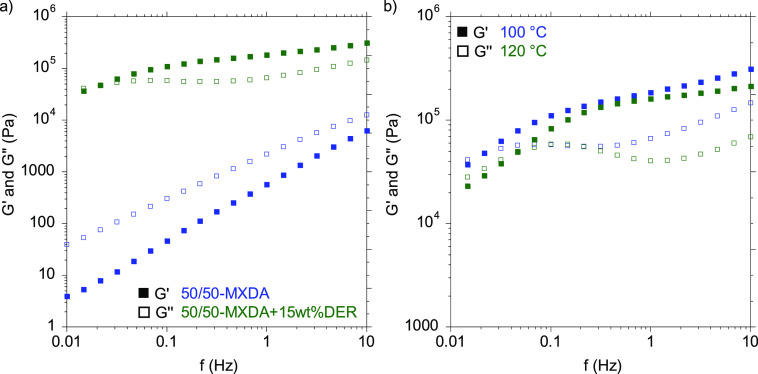
*G*′ and *G*′′ values from frequency sweep measurements for 50/50-MXDA (blue) and 50/50-MXDA+15wt%DER (green) at 100 °C.

Frequency sweep measurement of the 50/50MXDA+15DER as well as of the 50/50PXDA+15DER compositions at 100 °C demonstrated the restrictions in chain movements due to the addition of the epoxy hardener (Figure 9a and Figure S32, respectively). Nevertheless, at low frequencies, the crossover of *G*′ and *G*′′ was observed, meaning a transition from a solid-like to a liquid like behavior occurs, which allows the material to wet the substrate surface. In order to save time during application and enhance the wettability of the surfaces, adhesives were applied at 120 °C since the crossover of *G*′′ over *G*′ happens at shorter times at this temperature (Figure 9b).

Lap-shear strength and SAFT values on stainless steel are reported in Table 2. The addition of epoxy resin into the formulation endowed the materials with greater resistance to temperature, almost doubling the service temperatures. Moreover, as a signal of cohesive force reinforcement, adhesive failure in the PXDA-based adhesives was observed (Figure S32). On the contrary, the lap-shear strength decreased, probably due to the low temperature at which adhesives were applied near the SAFT. Nonetheless, values were within the typical lap-shear strengths for hot-melt adhesives, and reversibility of the bonding was demonstrated through similar, or even better, performance after two rebonding cycles. Adhesive failure was recorded after lap-shear tests, which confirmed the higher cohesiveness of the hybrid PHU adhesives as was expected due to the partially cross-linked nature (Figure S33).

**Table 2:** Lap-Shear Strength and Adhesion Failure Temperatures of the Enhanced PHU Hot-Melt Adhesives on Stainless Steel

		Lap-shear strength			
Code	Gel content (%)^a^	Original	Rebonding	Rebonding 2	SAFT (°C)
50/50-MXDA	b	7.0 ± 0.8	7.8 ± 0.8	8.5 ± 0.6	59 ± 1
50/50-MXDA+15 wt %DER	3.8 ± 2.8	3.1 ± 0.8	5.5 ± 1.1	8.9 ± 2.0	104 ± 4
50/50-PXDA	b	3.6 ± 1.0	6.2 ± 1.3	4.2 ± 0.9	63 ± 1
50/50-PXDA+15 wt %DER	41.3 ± 7.9	2.4 ± 0.8	3.1 ± 0.8	6.7 ± 2.3	119 ± 12

a Gel content after Soxhlet extraction in THF for 24 h.

b Samples dissolved totally in THF.

## 4 Conclusions

In this work, hot-melt adhesives based on PHUs have been prepared. We have elucidated their structural requirements to meet hot-melt adhesive properties. After a full structural characterization of PHUs by FTIR-ATR, ^1^H NMR, and rheological measurements, we found that the use of rigid diamines such as aromatic or cycloaliphatic ones is required to provide an adequate thermal transition. We hypothesize that these rigid diamines restrict the movement of polymer chains, reducing substantially the hydrogen bonding present in the PHU. The thermoreversible adhesion of the copolymers was successfully demonstrated by performing lap-shear measurements after two consecutive rebonding events. The adhesion performance was maintained in all cases when rigid diamines were used. While similar values in lap-shear strength were obtained for aromatic- and cycloaliphatic-based compositions, the shear adhesion failure temperatures (SAFTs) and shear resistance of the materials were superior when aromatic diamines were selected. Nevertheless, the service temperature was still not sufficient. In order to enhance the service temperature, cross-linked PHU hot-melt adhesives were prepared by adding a small amount of epoxy resin (D.E.R. 671, 15 wt %). The introduction of the epoxy resin endowed the adhesives with higher service temperatures, due to permanent cross-linking, without preventing the thermoreversibility. We believe that this study provides important insights for designing non-isocyanate polyurethane hot-melt adhesives.

## Supporting Information Available

^1^H NMR and FTIR-ATR spectra, AHEW, CEW, and EEW values, molar masses of PHU copolymers, rheology measurements, DSC and DMTA characterization, tack probe stress–strain curves, and nature of adhesive failure of adhesive joints of lap-shear, SAFT, and shear resistance (PDF)
