# Supplementary material for: Enhanced and Reusable Poly(hydroxy urethane)-Based Low Temperature Hot-Melt Adhesives
Source: ACS Polym Au. 2022 Jan 10;2(3):194–207. doi: 10.1021/acspolymersau.1c00053 (PMC9185748; doi:10.1021/acspolymersau.1c00053)
Supplement: Supplementary file 1 — lg1c00053_si_001.pdf [file lg1c00053_si_001.pdf]

Enhanced and Reusable Poly(Hydroxy Urethane)-Based Low-Temperature Hot-Melt Adhesives

Alvaro Gomez-Lopez,[ʒ] Naroa Ayensa,[ʒ] Bruno Grignard,[ψ] Lourdes Irusta,[ʒ] Iñigo Calvo,[¥] Alejandro J. Müller,[ʒ,γ] Christophe Detrembleur,[ψ] Haritz Sardon[ʒ]

[ʒ] POLYMAT and Department of Polymers and Advanced Materials: Physics, Chemistry and Technology, Faculty of Chemistry, University of the Basque Country UPV/EHU, Paseo Manuel de Lardizabal 3, 20018, Donostia-San Sebastián, Spain.

[ψ] Center for Education and Research on Macromolecules (CERM), CESAM Research Unit, University of Liège, allée du 6 août, Building B6A, Agora Square, 4000 Liège, Belgium.

[¥] ORIBAY Group Automotive S.L. R&D Department, Portuetxe bidea 18, 20018, Donostia-San Sebastián, Spain.

[γ] IKERBASQUE, Basque Foundation for Science, Plaza Euskadi 5, 48009, Bilbao, Spain

KEYWORDS. Non-isocyanate polyurethanes, poly(hydroxy urethane)s, hot-melt, adhesives, sustainability, green chemistry.

Titration of the cyclic carbonate by ^1H NMR.

A specified amount of cyclic carbonate (PPGdiCC or RdiCC) (around 50 mg) and standard solution of DMSO with toluene (around 30 mg of toluene dissolved in 5 mL of DMSO-d_6) were weighed into an NMR tube. The characteristic peaks of carbonate a, b and c (4.51, 4.29 and 4.92 ppm respectively) and CH_3 (2.32 ppm) of toluene were integrated. The integration of CH_3 of toluene was fixed to 300. Carbonate equivalent weight (CEW) of the carbonates was calculated according to eq S1, where m_{C5} – mass of cyclic carbonate introduced into the NMR tube, $n_{\text{function of carbonate}}$ – molar amount of function carbonate in cyclic carbonate, I_a, I_b, I_c – integrations of characteristics peaks a, b and c of carbonate, n_{toluene} – molar amount of toluene introduced in standard solution, I_{CH3} – integration of peak CH_3 of toluene. The CEW values for each cyclic carbonate were obtained in triplicate and are presented in Table S1.

$$CEW = \frac{m_{C5}}{n_{function\ of\ carbonate}} = \frac{m_{C5}\ x\ I_{CH3}}{(I_a + I_b + I_c)\ x\ n_{toluene}} \quad (S1)$$

Typical procedure for PHU homopolymer synthesis.

Homopolymerization of 100/0-MXDA was carried out by adding PPGdiCC (2.0196 g, 6.1 10^{-3} equiv) and MXDA (0.5160 g, 7.6 10^{-3} equiv) to a 25 mL round bottom flask. The mixture was placed into an oil bath at 80 °C and was left for 24 h under magnetic stirring. Afterwards, the reaction was cooled down and a viscous polymer was obtained. For the homopolymerization of 0/100-MXDA, RdiCC (1.1554 g, 6.1 10^{-3} equiv) and MXDA (0.5160 g, 7.6 10^{-3} equiv) were added to a 25 mL round bottom flask. The mixture was placed into an oil bath at 80 °C and was left for 24 h under magnetic stirring. After cooling down the reaction a yellowish hard homopolymer was obtained. Polymers were used without further purification.

Typical procedure for PHU copolymer synthesis.

PPGdiCC (11.1458 g, 33.5 10^{-3} equiv) and RdiCC (4.2520 g, 22.3 10^{-3} equiv) were added to a 100 mL jacketed glass reactor. The mixture was heated to 80 °C before adding MXDA (4.7472 g, 69.7 10^{-3} equiv). The reaction was left for 24 hours under continuous mechanical stirring at 200 rpm without using any catalyst. Afterwards, the reaction was cooled down and the polymer stored. Polymers were used without further purification.

Eq S2 was employed for the calculation of the amine mass. The molar ratio between PPGdiCC and RdiCC was fixed according to the different compositions and the molar ratio of amine to cyclic carbonates was fixed to 1.25 (r = 1.25). The number of active hydrogens of amine was fixed at 1 for one amine function reacting with one cyclic carbonate. The active hydrogen equivalent weight (AHEW) was calculated from eq S3.

$$m_{amine} = rAHEW_{amine}\left(\frac{m_{PPGdiCC}}{CEW_{PPGdiCC}} + \frac{m_{RdiCC}}{CEW_{RdiCC}}\right) \quad (S2)$$

$$AHEW = \frac{AEW}{number\ of\ active\ hydrogen} \quad (S3)$$

Typical procedure for enhanced hot-melt adhesive preparation.

Around 4 g of 50/50-MXDA or 50/50-PXDA composition were heated up to 90 °C and mixed with D.E.R.™ 671 (0.6 g, 15 wt%) for 20 minutes. Afterwards, the sample was kept in an oven at 55 °C for 3 days to assure complete reaction of the amine with epoxy groups.

Table S1. Carbonate equivalent weight (CEW), amine equivalent weight (AEW), epoxy equivalent weight (EEW) and thiol equivalent weight (TEW) values.

Reactant	CEW (g equiv^{-1})	AEW (g equiv^{-1})	EEW (g equiv^{-1})
PPGdiCC	190 ± 5	-	-
RdiCC	190 ± 5	-	-
BdiCC	167 ± 4	-	-
MXDA	-	68.10	-
PXDA	-	68.10	-
CBMA	-	71.12	-
HMDA	-	58.10	-
1,12-DAD	-	100.18	-
D.E.R.™ 671	-	-	475-550[a]

[a] value provide by Dow Chemical Company. The media between values was employed for calculations.

Synthesis of PHU homopolymers from RdiCC and MXDA

The completion of the reaction was followed by FTIR-ATR through the disappearance of the carbonyl stretching vibration of the carbonate at 1782 cm^{-1} and the appearance of the carbonyl stretching vibration band of the urethane at 1694 cm^{-1} (Figure S1). Additionally, common signals at 3320 cm^{-1} (-OH, $-NH_2$ stretching), 2930-2970 cm^{-1} ($-CH_2$, $-CH_3$ stretching), at 1592, 1491 and 1450 cm^{-1} (C=C stretching of the aromatic rings), at 1531 cm^{-1} (N-H bending), at 1240 cm^{-1} (C-O-C stretching), at 1038 cm^{-1} (C-O stretching) and at 775 and 700 cm^{-1} (C-H out of plane bending vibrations of the aromatic rings) confirmed the structure of the homopolymer. ^{1}H-NMR spectrum of the MXDA-RdiCC PHU is shown in (Figure S2). The disappearance of the signals of the cyclic carbonate (5.02, 4.62-4.53 and 4.23-4.13 ppm) and the appearance of the signals at 7.72 (-N<u>H</u>-C(O)O-, urethane) and at 4.16 ppm (methylene group of the amine), further confirmed the formation of the RdiCC-based homopolymer.

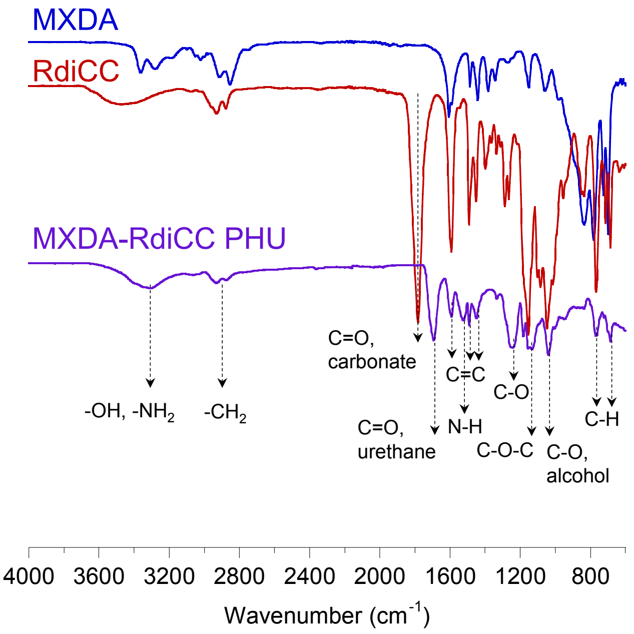

Figure S1. FTIR-ATR spectra of MXDA-RdiCC PHU together with the respective starting monomers.

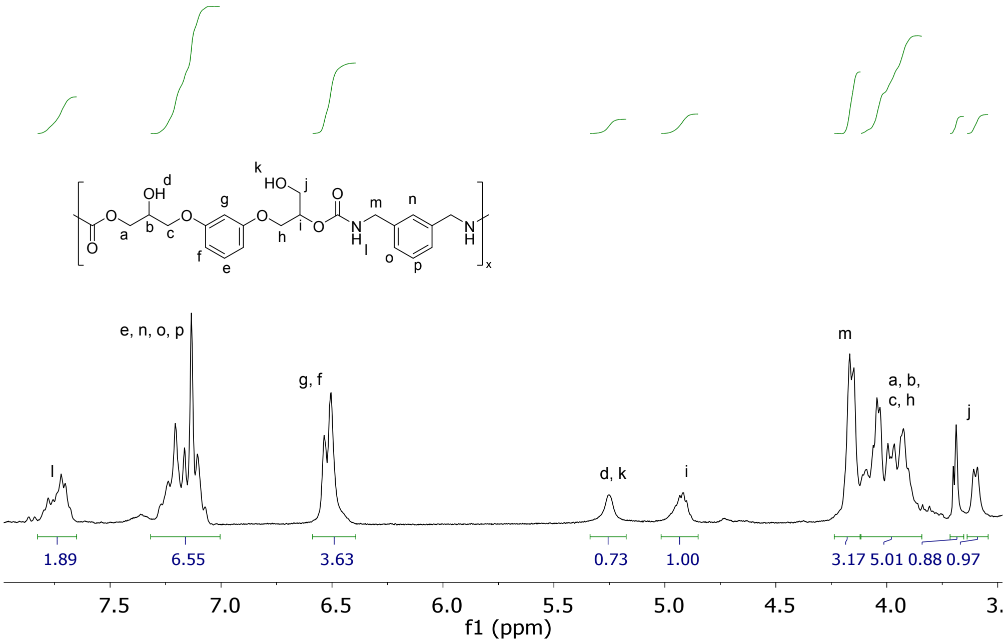

Figure S2. ^{1}H NMR spectrum of the MXDA-RdiCC PHU homopolymer.

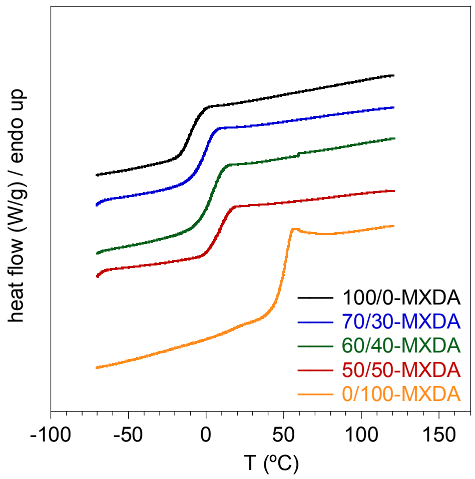

Figure S3. DSC thermograms of the PHU based on different PPGdiCC/RdiCC molar ratios and MXDA.

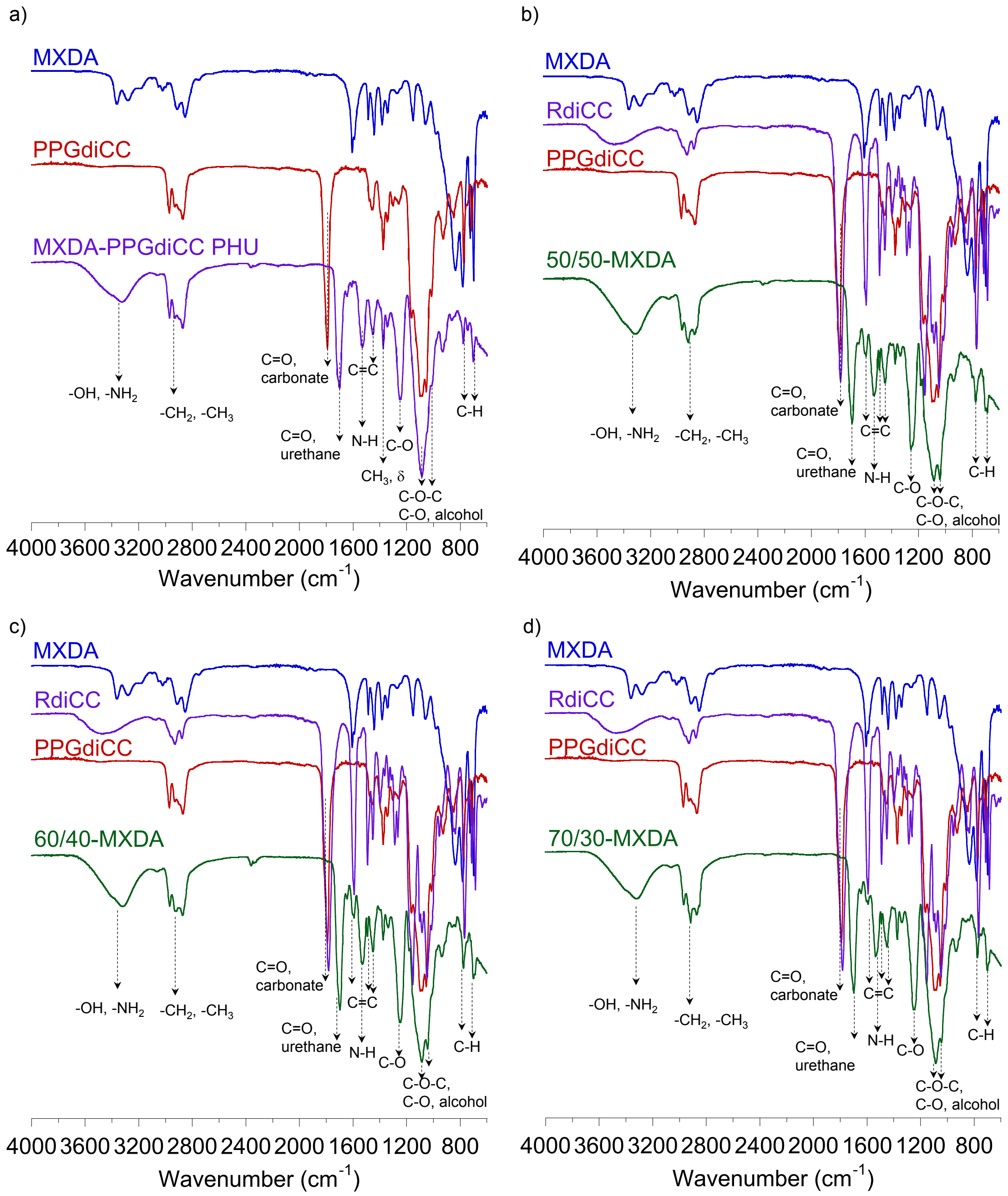

Figure S4. FTIR-ATR spectra of a) MXDA-PPGdiCC PHU, b)50/50-MXDA, c) 60/40-MXDA and d) 70/30-MXDA compositions. MXDA, RdiCC and PPGdiCC spectra were added for a clearer comparison of the new bands after reaction.

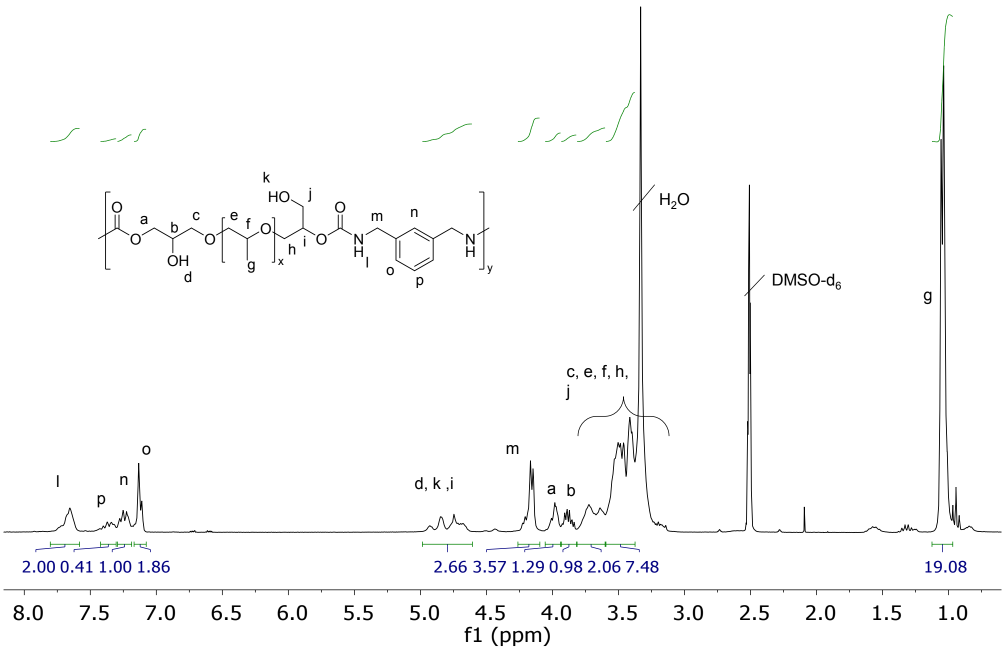

Figure S5. NMR spectrum of the MXDA-PPGdiCC PHU homopolymer.

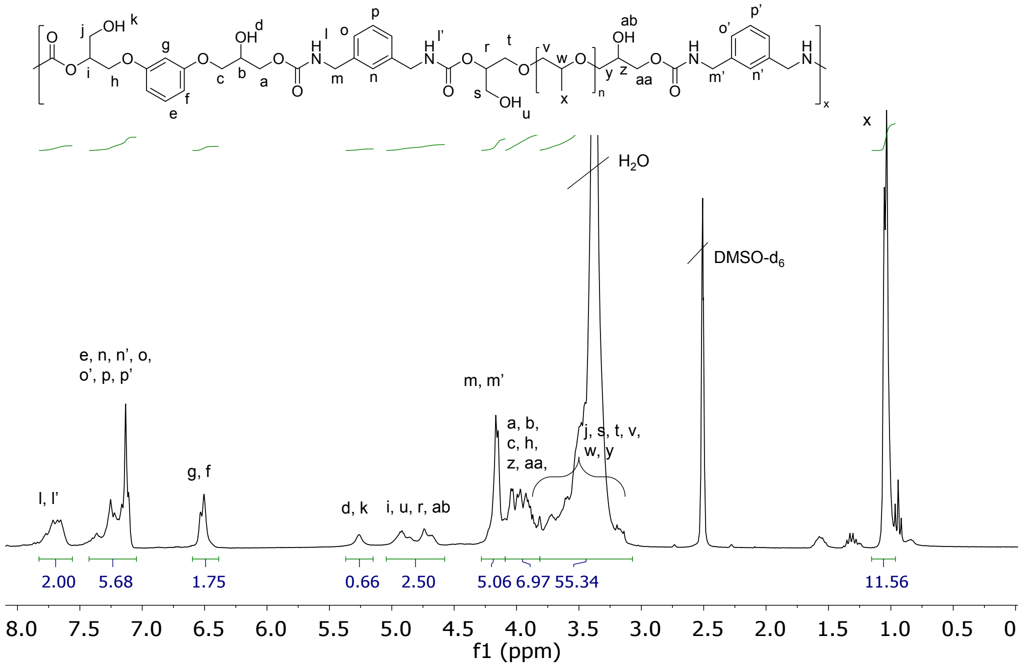

Figure S6. ^{1}H NMR spectrum of the 50/50-MXDA copolymer.

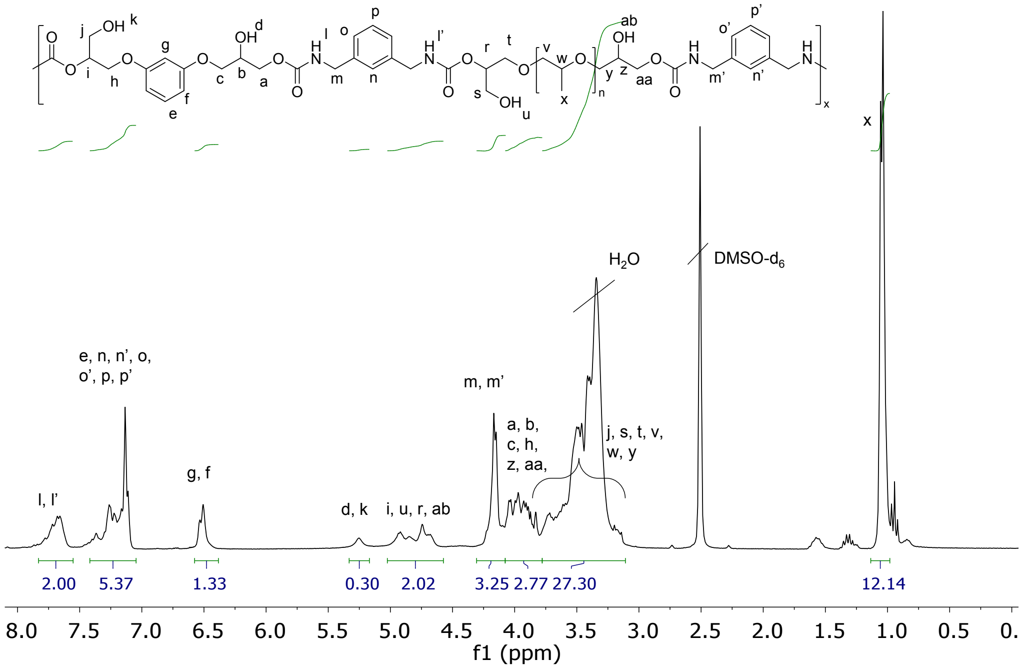

Figure S7. ^{1}H NMR spectrum of the 60/40-MXDA copolymer.

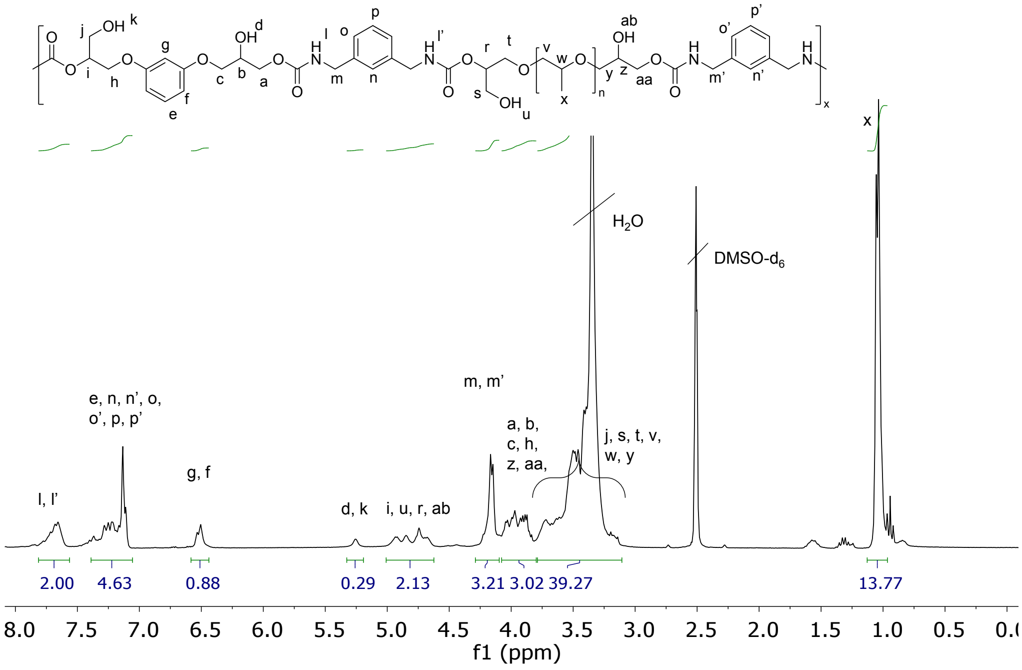

Figure S8. ^{1}H NMR spectrum of the 70/30-MXDA copolymer.

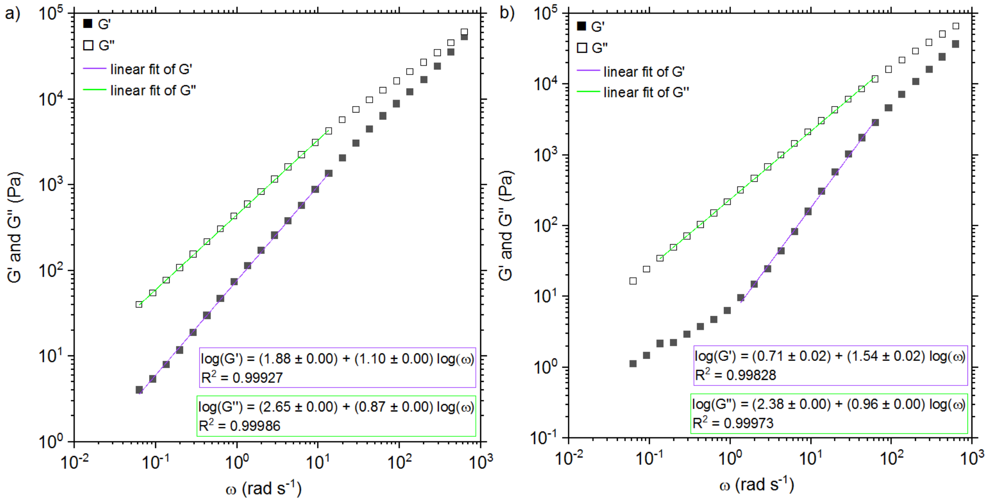

Figure S9. G' and G'' values as a function of ω at 100 °C of a) 50/50-MXDA and b) 70/30-MXDA with the corresponding linear fit. Equations of the linear fit are presented in the boxes.

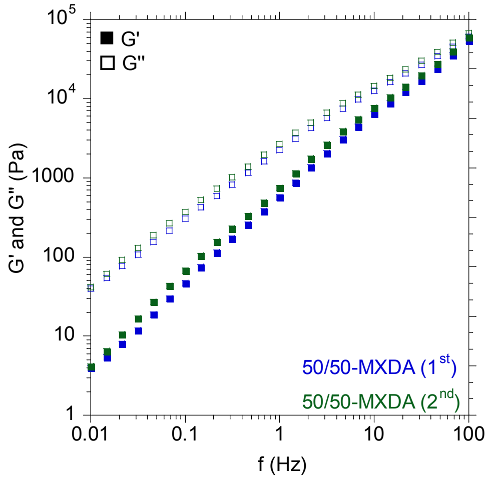

Figure S10. G' and G'' values between 0.01 and 100 Hz at a) 10 °C, b) 25 °C, c) 50 °C and d) 80 °C of 70/30-MXDA (blue) and 50/50-MXDA (green).

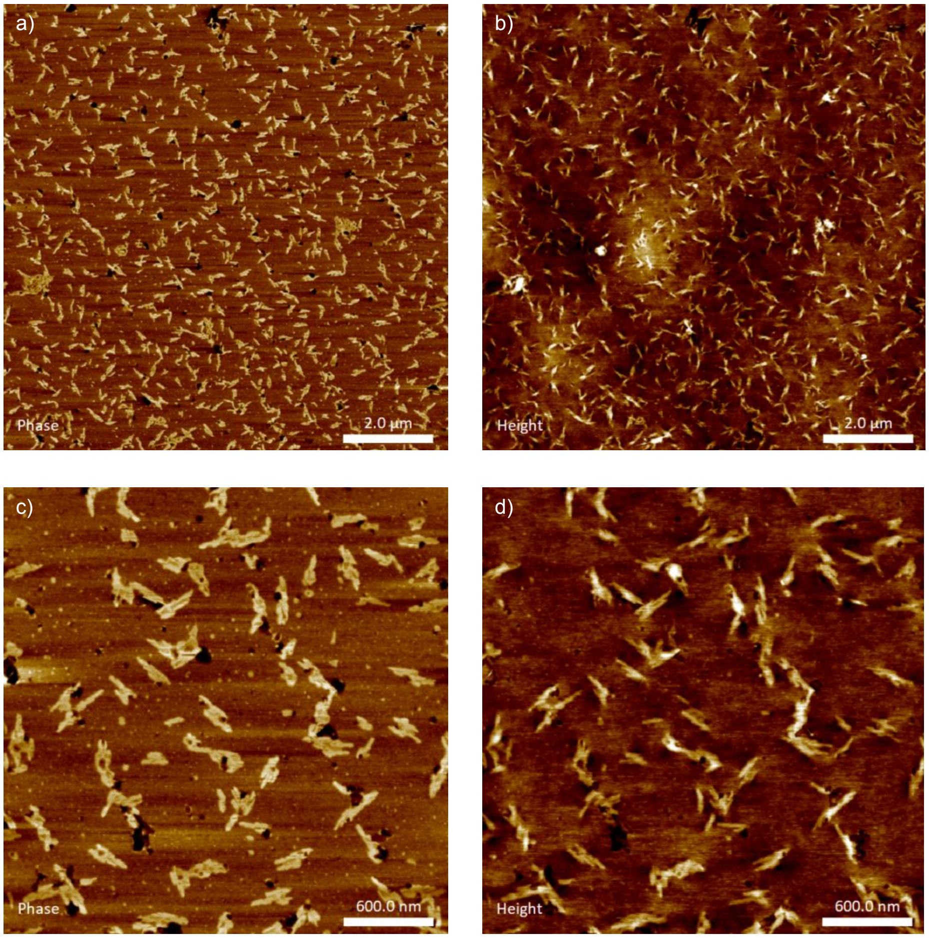

Figure S11. Phase and height AFM images of 50/50-MXDA at two different scales.

Synthesis of copolymer based on PPGdiCC, BdiCC and MXDA (50/50-BdiCC-MXDA

After 24 h, the completion of the reaction was confirmed by the total disappearance of the C=O stretching band of the cyclic carbonates (1790 cm-1, PPGdiCC and 1780 cm-1, BdiCC) in the FTIR-ATR spectrum (Figure S11). Further analysis by 1H NMR characterization showed the signals at 7.6-7.8 ppm of the –NH-C(O)O and the signal of -CH2NH- at 4.15 and 4.17 ppm, verifying the formation of the urethane linkage (Figure S12).

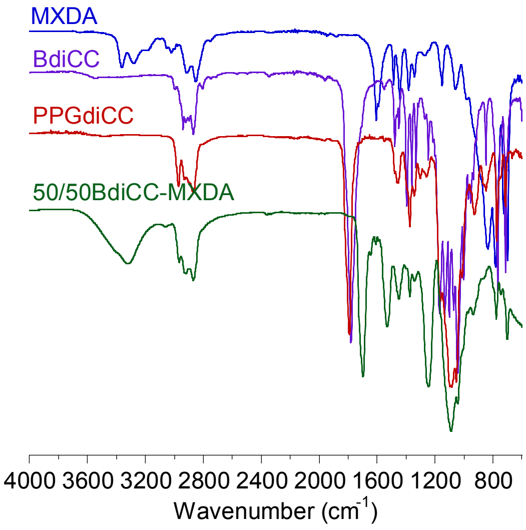

Figure S12. FTIR-ATR spectrum of 50/50BdiCC compared to the initial monomer spectra of MXDA, BdiCC and PPGdiCC.

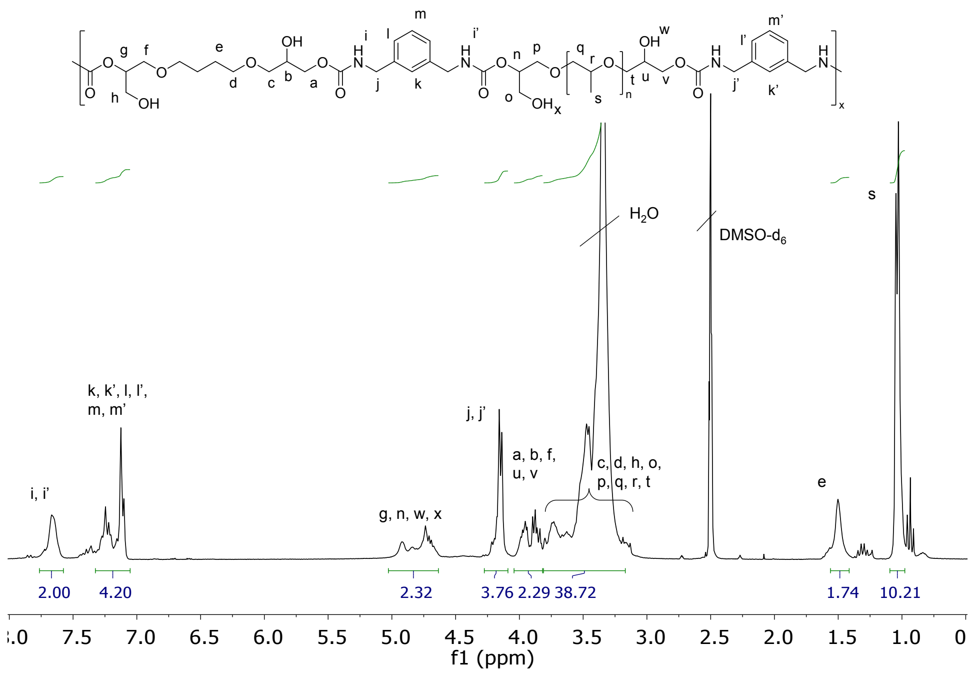

Figure S13. ^{1}H NMR spectrum of 50/50BdiCC-MXDA composition.

Table S2. Average molar masses and glass transition temperatures of the PHU-based hot-melt adhesives.

Composition PPGdiCC/RdiCC (mol%)	Diamine	Code	M_n[a] kg mol^{-1}	M_w[a] kg mol^{-1}	Đ[a]	T_g (°C)	GC[e] (%)
70/30	MXDA	70/30-MXDA	2.8 ± 0.0	6.4 ± 0.2	2.3 ± 0.0	10.3[c]	[f]
60/40		60/40-MXDA	2.5 ± 0.2	6.3 ± 0.4	2.5 ± 0.0	20.4[c]	[f]
50/50		50/50-MXDA	2.6 ± 0.1	6.4 ± 0.3	2.5 ± 0.0	30.5[c]	[f]
*		50/50BdiCC-MXDA	2.5 ± 0.0	5.8 ± 0.1	2.3 ± 0.0	15.5[c]	[f]
	PXDA	50/50-PXDA	3.5 ± 0.7	7.5 ± 0.3	2.2 ± 0.4	26.5[c]	[f]
	CBMA	50/50-CBMA	1.9 ± 0.0	4.3 ± 0.1	2.2 ± 0.0	30.5[c]	[f]
	HMDA	50/50-HMDA	[b]	[b]	[b]	4.9[d]	65.0 ± 5.0
	1,12-DAD	50/50-DAD	[b]	[b]	[b]	7.2[d]	60.7 ± 2.2
	PXDA**	-	5.6 ± 1.0	9.9 ± 0.8	1.8 ± 0.2	[g]	[f]

[a] Average molar masses based on calibration with polystyrene standards ranging from 573 to 3,848,000 g mol^{-1}; [b] not determined because samples were not soluble in THF; T_g values from [c] rheological temperature sweep measurements done in parallel plates and [d] from dynamic mechanical temperature analysis measurements (Figure S18); [e]gel content after Soxhlet extraction in refluxing THF for 24 h; [f]soxhlet extraction was not performed since samples presented total solubility in THF (no gel content); [g] not determined. * RdiCC was substituted by BdiCC for this formulation. ** Cyclic carbonate to amine stoichiometric balanced reaction.

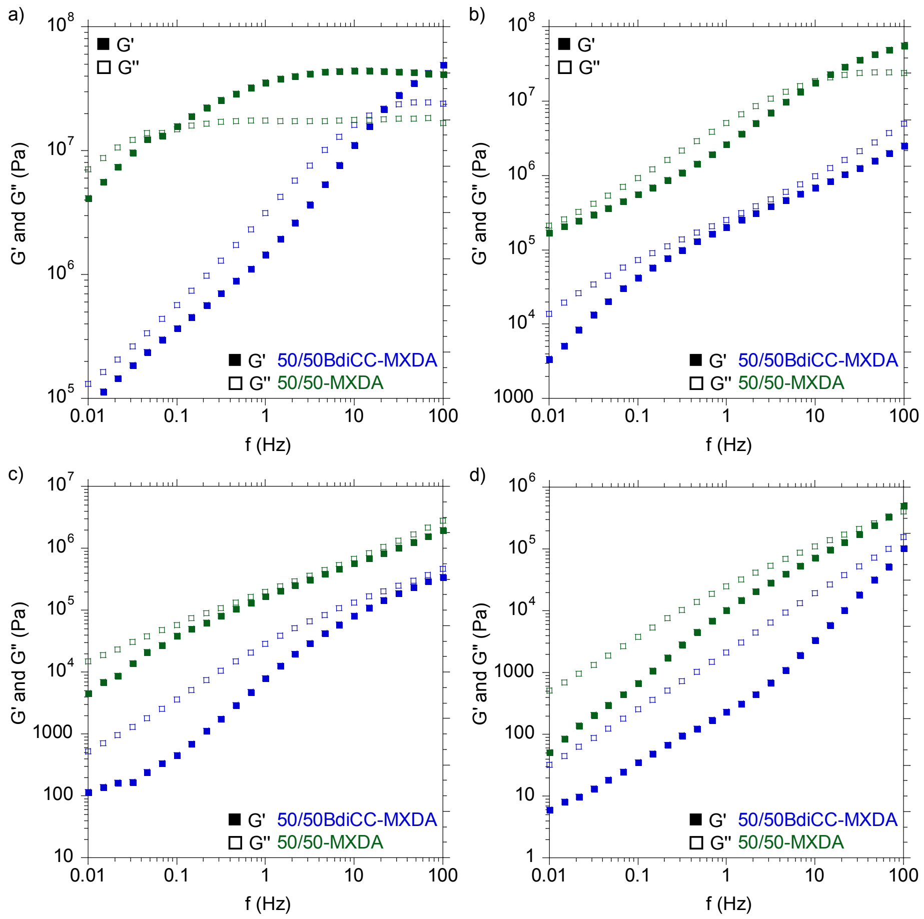

Figure S14. G’ and G’’ values between 0.01 and 100 Hz of 50/50BdiCC-MXDA and 50/50-MXDA compositions at a) 10 °C b) 25 °C c) 50 °C d) 80 °C.

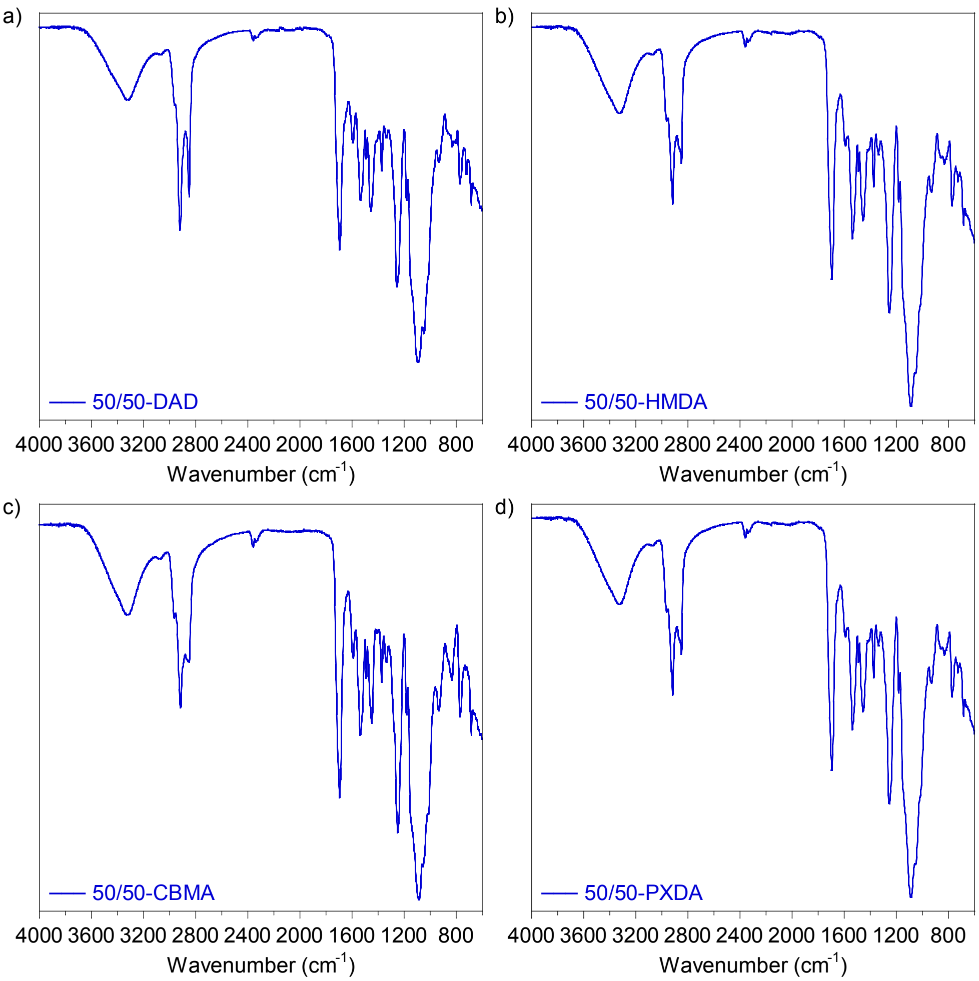

Figure S15. FTIR spectra of a) 50/50-DAD, b) 50/50-HMDA, c) 50/50-CBMA and d) 50/50-PXDA compositions.

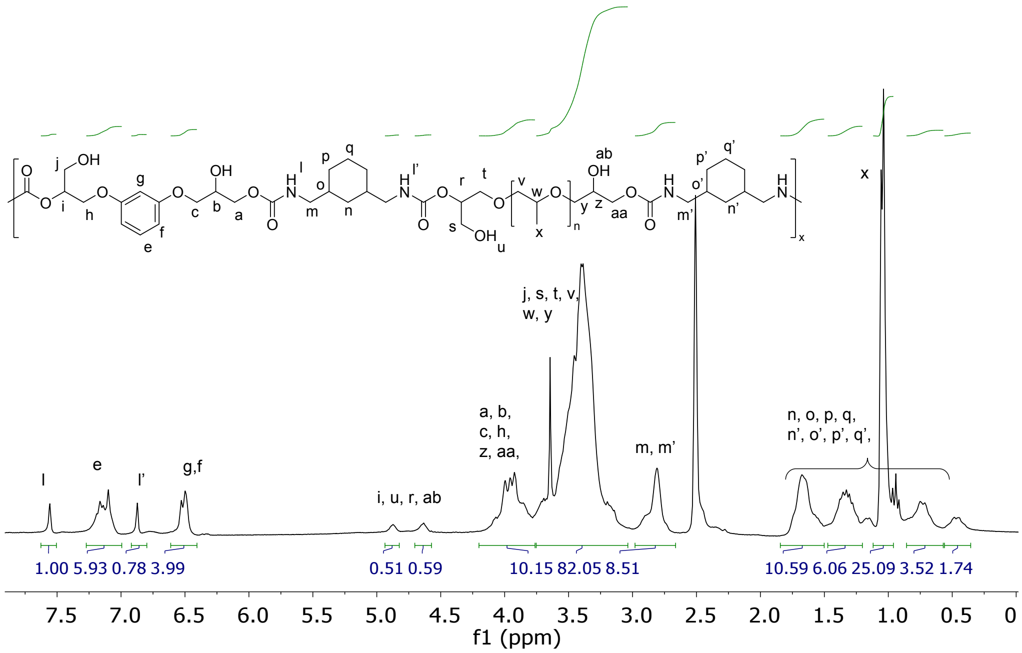

Figure S16. NMR spectrum of 50/50-CBMA.

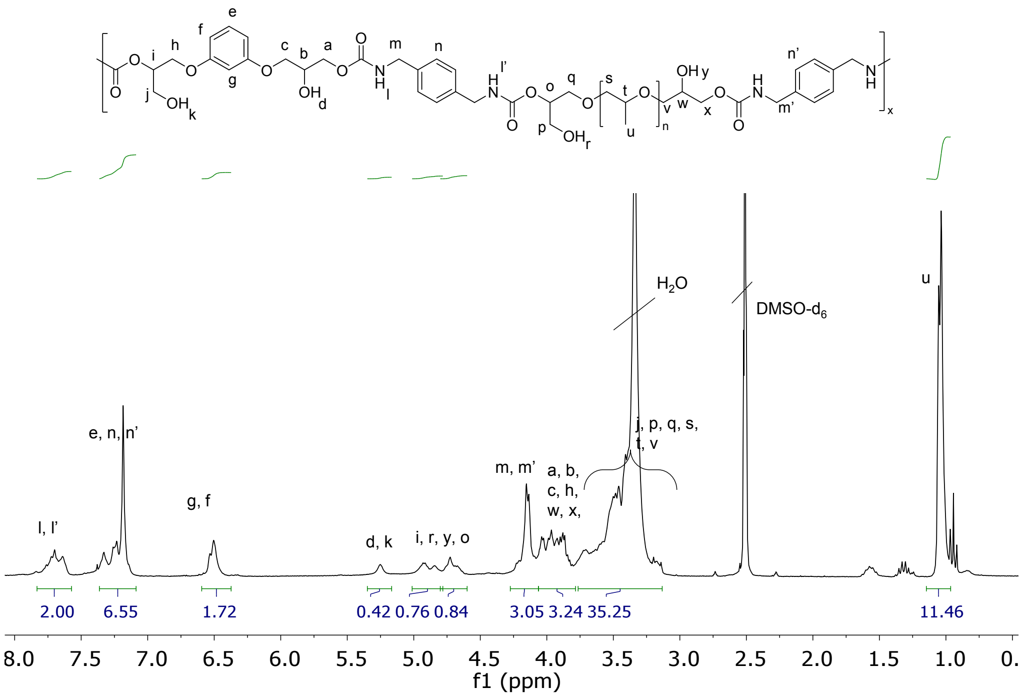

Figure S17. NMR spectrum of 50/50-PXDA.

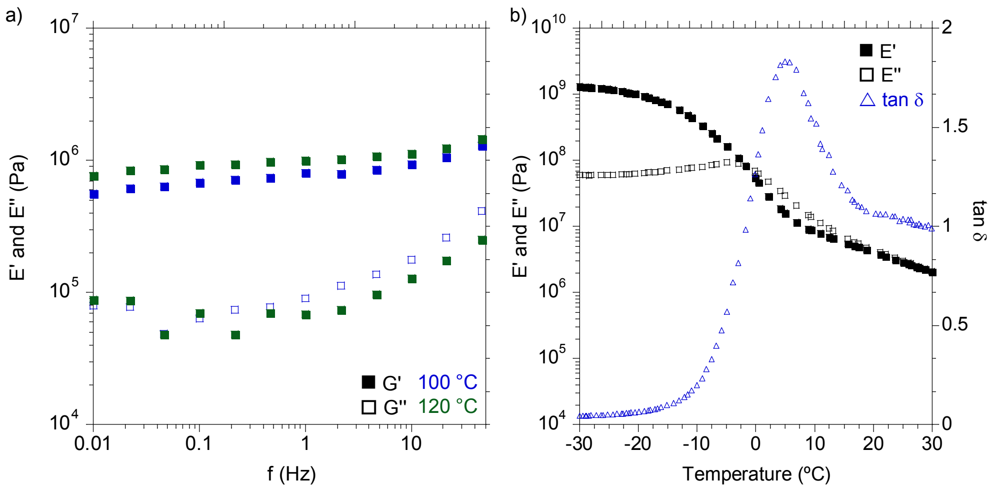

Figure S18. a) E' and E'' values between 0.01 and 50 Hz of 50/50-DAD at 100 (blue) and 120 (green) °C; b) E', E'' and tan [illegible] values of 50/50-HMDA from DMTA analysis. DMTA was employed to determine the glass transition temperature of the material.

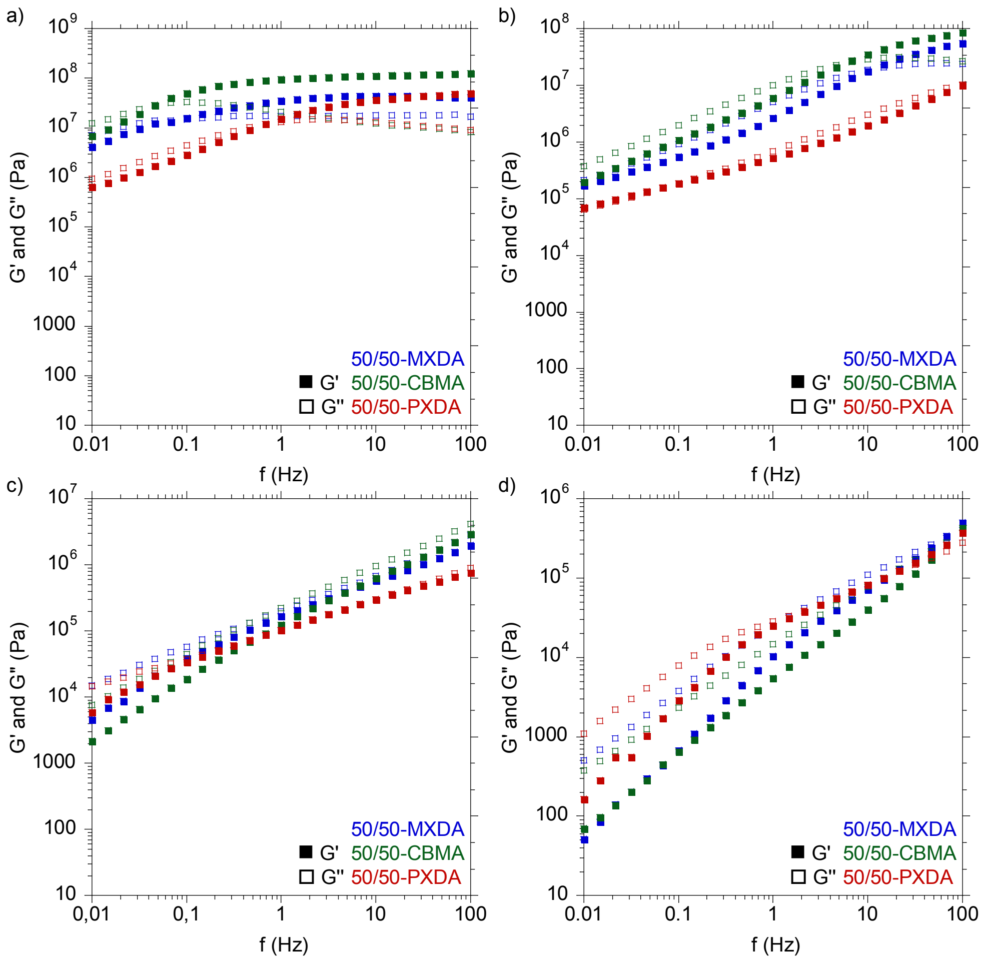

Figure S19. G’ and G’’ values between 0.01 and 100 Hz of 50/50-MXDA, 50/50-CBMA and 50/50-PXDA compositions at a) 10 °C, b) 25 °C, c) 50 °C and d) 80 °C.

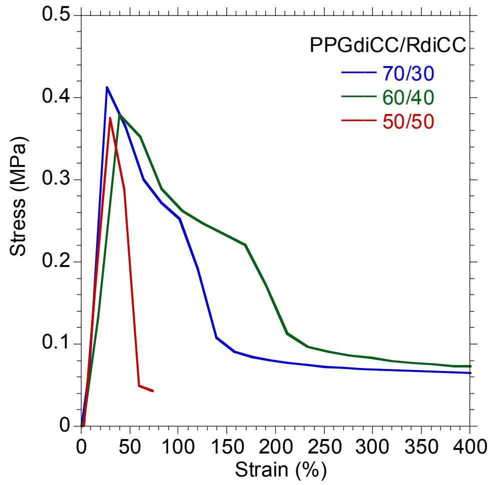

Figure S20. Probe tack stress-strain curves for 70/30-, 60/40- and 50/50-MXDA compositions at 80 °C.

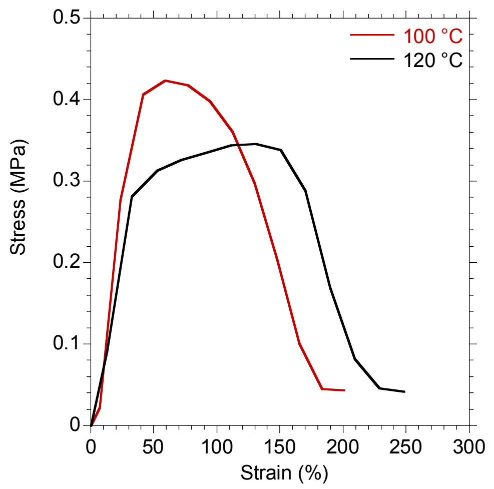

Figure S21. Probe tack stress-strain cures of 50/50-PXDA at 100 °C and 120 ºC.

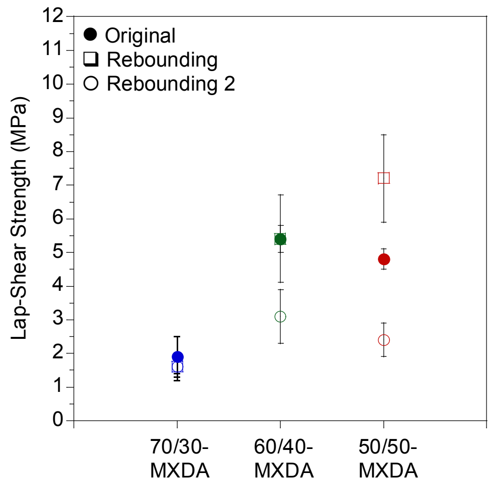

Figure S22. Lap-shear strength values for 70/30-, 60/40- and 50/50-MXDA formulations when samples were applied onto the substrates at 80 °C

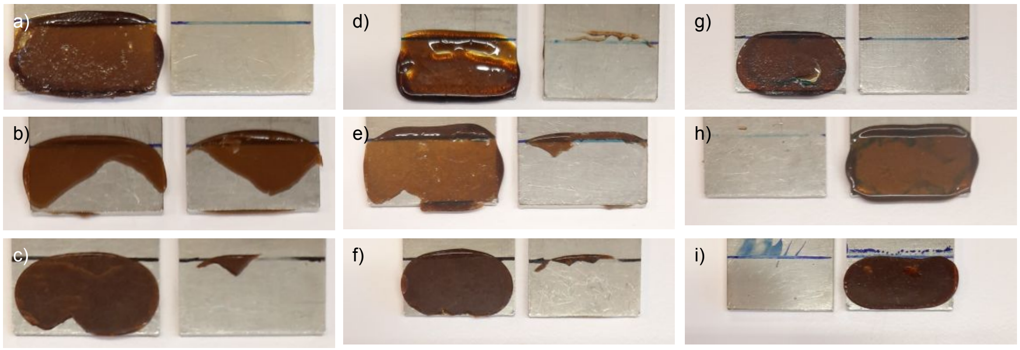

Figure S23. Photos of the representative adhesion failure of a) 70/30-MXDA; b) 60/40-MXDA and c) 50/50-MXDA applied at 80 °C; d) 70/30-MXDA; e) 60/40-MXDA and f) 50/50-MXDA after re-bonding at 80 °C; g) 70/30-MXDA; h) 60/40-MXDA and i) 50/50-MXDA and after a second re-bonding at 80 °C.

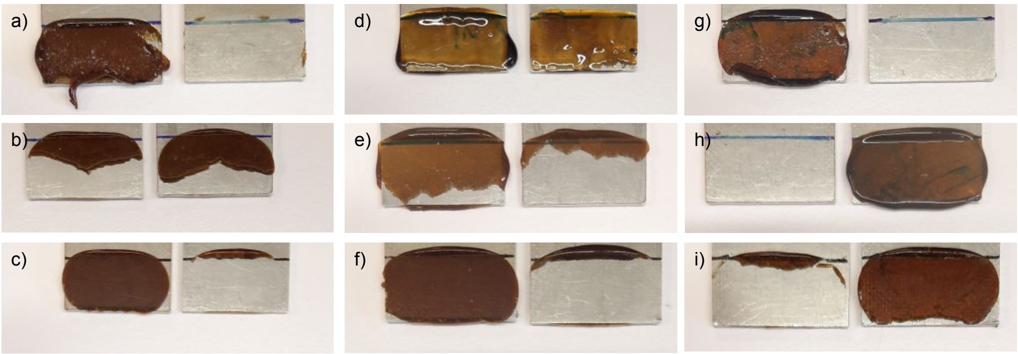

Figure S24. Photos of the representative adhesion failure of a) 70/30-MXDA; b) 60/40-MXDA and c) 50/50-MXDA applied at 100 °C; d) 70/30-MXDA; e) 60/40-MXDA and f) 50/50-MXDA after re-bonding at 100 °C; g) 70/30-MXDA; h) 60/40-MXDA and i) 50/50-MXDA after a second re-bonding at 100 °C.

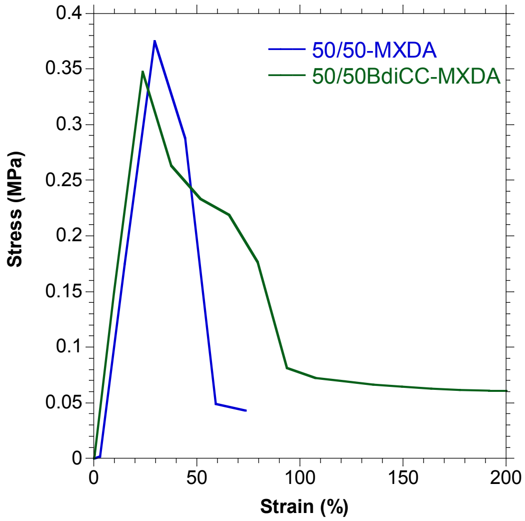

Figure S25. Stress-strain curves for 50/50BdiCC-MXDA and 50/50-MXDA compositions at 80 °C. 50/50-MXDA curve is depicted for easier comparison.

Table S3. Lap-shear strength, SAFT and shear resistance values for 50/50BdiCC-MXDA.

Code	Lap-Shear Strength (MPa)			SAFT (°C)	Shear Resistance (min)
	initial	Re-bonding	Re-bonding 2		
50/50-MXDA*	7.0 ± 0.8	7.8 ± 0.8	8.5 ± 0.6	59 ± 1	371 ± 18
50/50BdiCC-MXDA	2.0 ±0.4	0.5 ± 0.1	1.2 ± 0.1	35 ± 0	9 ± 0

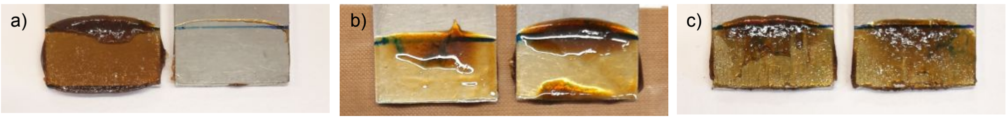

Figure S26. Photos of the representative adhesion failure of 50/50BdiCC-MXDA a) applied at 100 °C, b) after a re-bonding at 100 °C and c) after a second re-bonding at 100 °C.

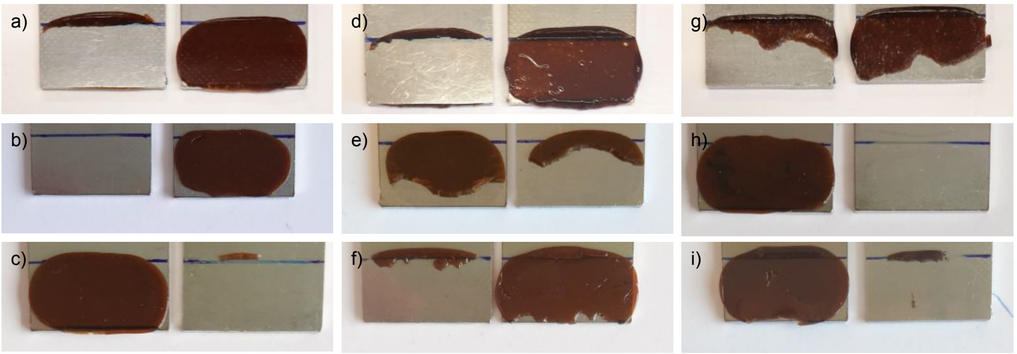

Figure S27. Photos of the representative adhesion failure of a) 50/50-CBMA and b) 50/50-PXDA applied at 100 °C; c) 50/50-PXDA applied at 120 °C; d) 50/50-CBMA and e) 50/50-PXDA after a re-bonding at 100 °C; f) 50/50-PXDA after a re-bonding at 120 °C; g) 50/50-CBMA and h) 50/50-PXDA after a second re-bonding at 100 °C; i) 50/50-PXDA after a second re-bonding at 120 °C.

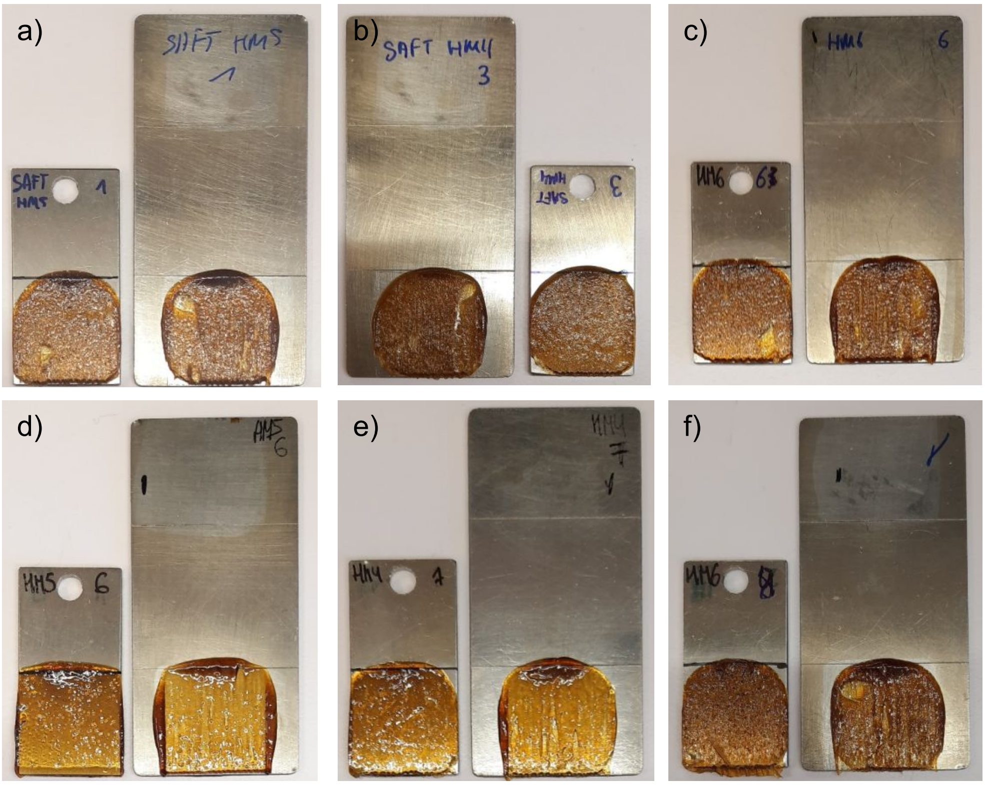

Figure S28. Photos of the representative adhesion failure of a) 70/30-MXDA; b) 60/40-MXDA and c) 50/50-MXDA after SAFT tests and d) 70/30-MXDA; e) 60/40-MXDA and f) 50/50-MXDA after shear resistance measurements.

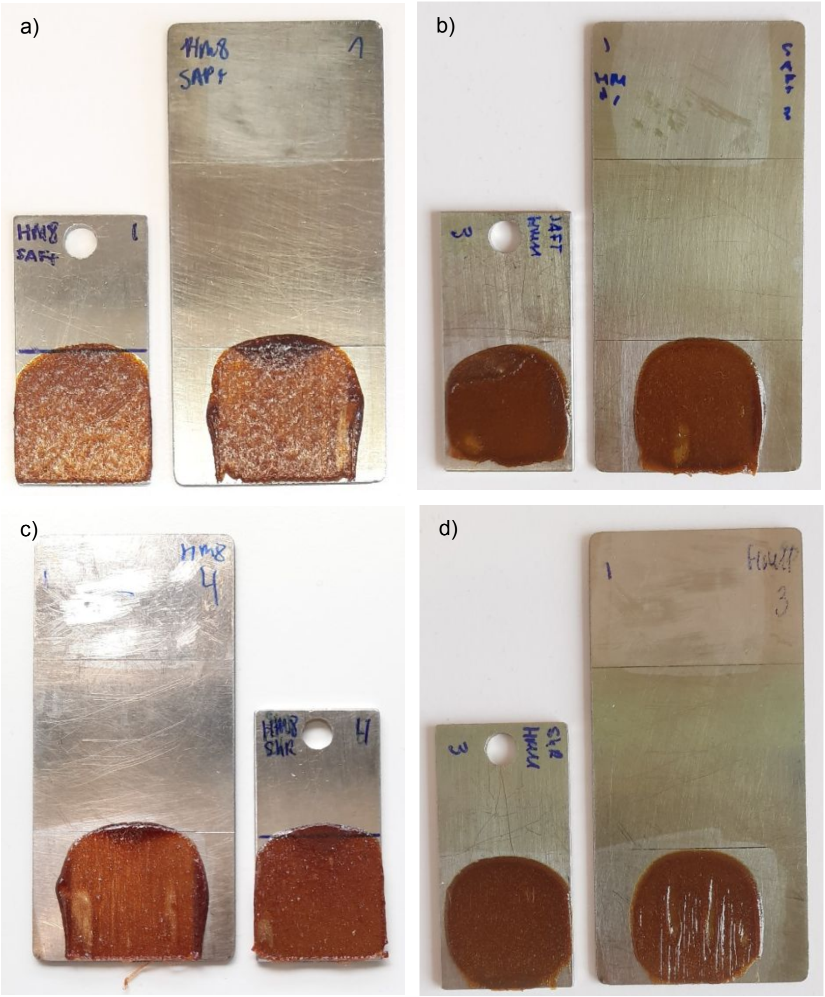

Figure S29. Photos of the representative adhesion failure of a) 50/50-CBMA and b) 50/50-PXDA after SAFT tests and c) 50/50-CBMA and d) 50/50-PXDA after shear resistance measurements.

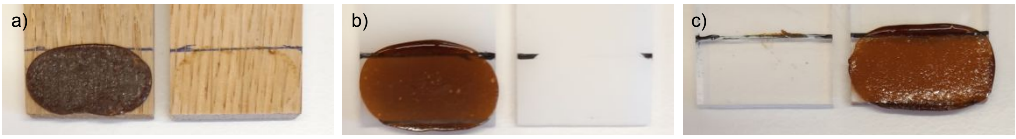

Figure S30. Photos of the representative adhesion failure after lap-shear tests of 50/50-MXDA when it was applied onto a) oak, b) PE-HD and c) PMMA.

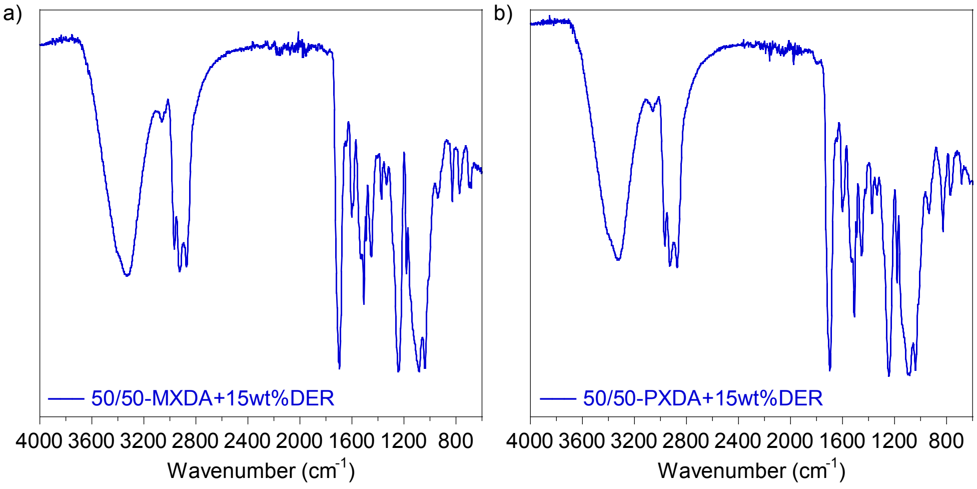

Figure S31. FTIR spectra of a) 50/50-MXDA+15wt%DER and b) 50/50-PXDA+15wt%DER compositions.

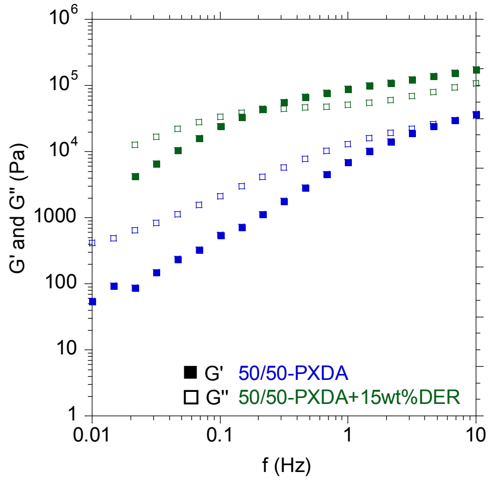

Figure S32. Photos of the representative adhesion failure after lap-shear tests of a) 50/50-MXDA+15DER and b) 50/50-PXDA+15DER applied at 120 °C; c) 50/50-MXDA+15DER and d) 50/50-PXDA+15DER after re-bonding at 120 °C; e) 50/50-MXDA+15DER and f) 50/50-PXDA+15DER after a second re-bonding at 120 °C.

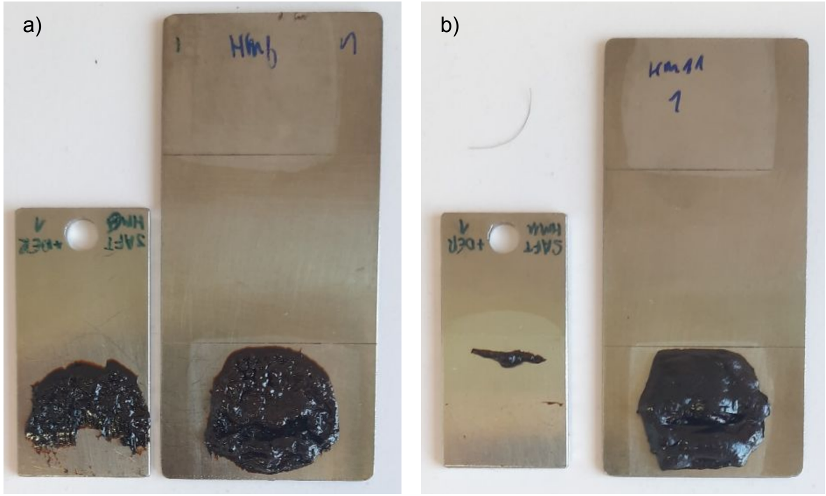

Figure S33. Photos of the representative adhesion failure of a) 50/50-MXDA+15DER and b) 50/50-PXDA+15DER after SAFT tests.

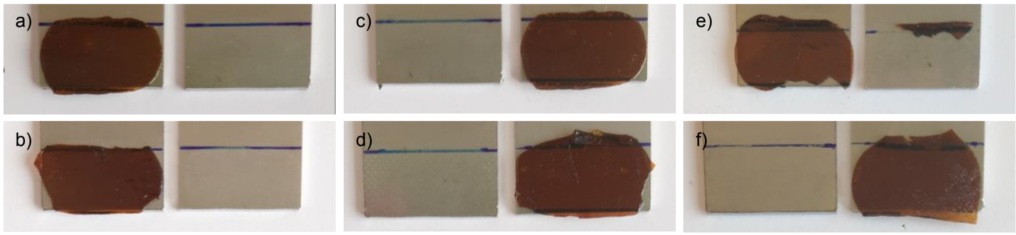

Figure S34. Photos of the representative adhesion failure after lap-shear tests of a) 50/50-MXDA+15DER and b) 50/50-PXDA+15DER applied at 120 °C; c) 50/50-MXDA+15DER and d) 50/50-PXDA+15DER after re-bonding at 120 °C; e) 50/50-MXDA+15DER and f) 50/50-PXDA+15DER after a second re-bonding at 120 °C.
